# Functional and Structural Investigation of Chalcone Synthases Based on Integrated Metabolomics and Transcriptome Analysis on Flavonoids and Anthocyanins Biosynthesis of the Fern *Cyclosorus parasiticus*

**DOI:** 10.3389/fpls.2021.757516

**Published:** 2021-10-28

**Authors:** Meng Niu, Jie Fu, Rong Ni, Rui-Lin Xiong, Ting-Ting Zhu, Hong-Xiang Lou, Peng Zhang, Jianxu Li, Ai-Xia Cheng

**Affiliations:** ^1^Key Laboratory of Chemical Biology of Natural Products, Ministry of Education, School of Pharmaceutical Sciences, Shandong University, Jinan, China; ^2^National Key Laboratory of Plant Molecular Genetics, Center for Excellence in Molecular Plant Sciences, Institute of Plant Physiology and Ecology, Shanghai Institutes for Biological Sciences, Chinese Academy of Sciences, Shanghai, China

**Keywords:** metabolomics, transcriptomics, flavonoids biosynthesis, chalcone synthase, *Cyclosorus parasiticus*

## Abstract

The biosynthesis of flavonoids and anthocyanidins has been exclusively investigated in angiosperms but largely unknown in ferns. This study integrated metabolomics and transcriptome to analyze the fronds from different development stages (S1 without spores and S2 with brown spores) of *Cyclosorus parasiticus*. About 221 flavonoid and anthocyanin metabolites were identified between S1 and S2. Transcriptome analysis revealed several genes encoding the key enzymes involved in the biosynthesis of flavonoids, and anthocyanins were upregulated in S2, which were validated by qRT-PCR. Functional characterization of two chalcone synthases (CpCHS1 and CpCHS2) indicated that CpCHS1 can catalyze the formation of pinocembrin, naringenin, and eriodictyol, respectively; however, CpCHS2 was inactive. The crystallization investigation of CpCHS1 indicated that it has a highly similar conformation and shares a similar general catalytic mechanism to other plants CHSs. And by site-directed mutagenesis, we found seven residues, especially Leu199 and Thr203 that are critical to the catalytic activity for CpCHS1.

## Introduction

**Figure 1:**
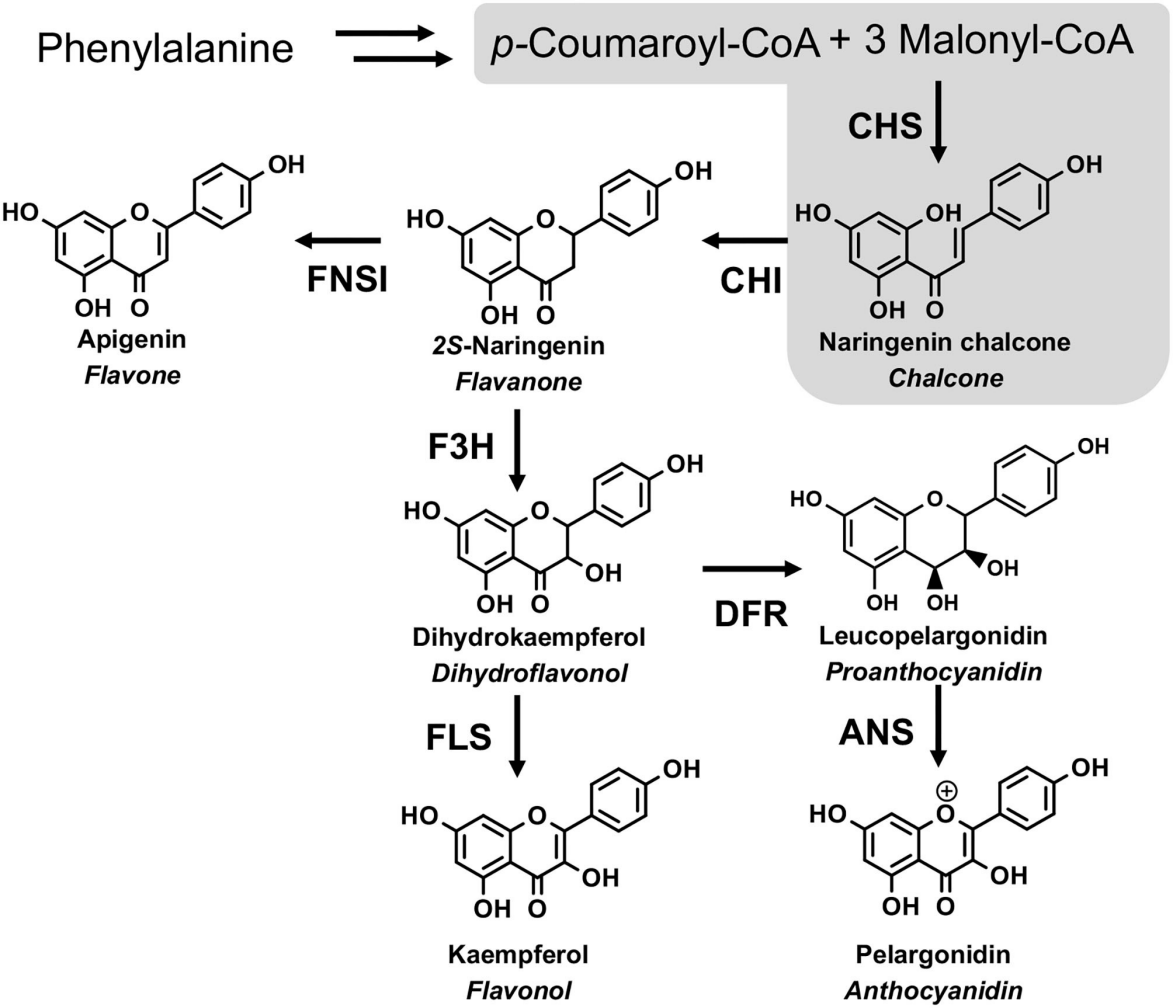
The flavonoid biosynthesis pathway. CHS, chalcone synthase; CHI, chalcone isomerase; FNS I, flavone synthase I; F3H, flavanone-3-hydroxylase; FLS, flavonol synthase; DFR, dihydroflavonols 4-reductase; ANS, anthocyanidin synthase. The gray area is the focus of this study.

Flavonoids are ubiquitously distributed in the plant kingdom and have diverse functions in plants, including protecting plants against UV-B-induced damage, attracting pollinators, playing as pigments, mediating the interaction of the plant with insects, or microbe (Martens and Mithofer, 2005; Righini et al., 2019). Flavonoids also show various pharmaceutical activities, including antioxidant and anti-inflammatory (Martens and Mithofer, 2005). The biosynthesis of flavonoids starts with the condensation of one molecule of *p-*coumaryl CoA with three molecules of malonyl-CoA through the catalysis of chalcone synthase (CHS) to generate naringenin chalcone. Then, chalcone isomerase (CHI) catalyzes the intramolecular and stereospecific cyclization of naringenin chalcone to form naringenin. Flavanone 3-hydroxylase (F3H) can catalyze naringenin to form dihydroflavonol. Subsequently, flavonol synthase (FLS) converts dihydroflavonols to their corresponding flavonols. Alternatively, dihydroflavonols were catalyzed by dihydroflavonol 4-reductase (DFR) and anthocyanidin synthase (ANS) to form anthocyanins (Figure 1).

Chalcone synthase, a well-studied ubiquitous plant-specific type III polyketide synthases (PKSs), is the first key enzyme in the flavonoid biosynthetic pathway. Since the first isolation of the CHS gene from parsley (*Petroselium hortense*) in 1983 (Reimold et al., 1983), more than 20 functionally different CHSs have been cloned and characterized in various plants (Abe and Morita, 2010; Liou et al., 2018; Yonekura-Sakakibara et al., 2019). The crystal structure *Medicago sativa* CHS2 was solved, which shares 74% sequence identity with the *Gerbera hybrida* 2-pyrone synthase (2PS) (Abe and Morita, 2010). Crystallographic and biochemistry studies on *Medicago sativa* CHS2 and 2PS (Jez et al., 2000) clearly demonstrate that the volume and the shape of the catalytic pocket principally influence the functional diversity of type III PKSs (Abe and Morita, 2010). Meanwhile, the crystallographic analysis revealed that the overall structure of *Freesia hybrida* CHS1 (Sun et al., 2015) and *Arachis hypogaea* (peanut) stilbenesynthase (STS) (Shomura et al., 2005) are highly homologous to that of alfalfa CHS. And a close-up view of the soybean CHS active site showed that the orientations of catalytic residues (Cys164, His302, and Asn335) are similar to other CHSs, with the electron densities for Cys164 side chains being in their doubly oxidized forms (Imaizumi et al., 2020). Weng et al. found that the doubly oxidized catalytic cysteine sulfinic acid is widely observed in the crystal structures of CHS from euphyllophytes (ferns and seed plants), but not from either lycophyte or moss species (Liou et al., 2018). Based on the structural and biochemistry studies of type III PKS, the catalytic mechanism of CHS enzyme was proposed. The reaction initiated from loading the starter molecule *p*-coumaroyl-CoA onto the catalytic cysteine, which also serves as the attachment site of the growing polyketide chain during the iterative elongation steps. This initial reaction step requires the cysteine to be present as a thiolate anion before loading of the starter molecule, followed by iterative decarboxylative Claisen-type condensations of malonyl-CoA and the final cyclization of the enzyme-bound poly-β-keto intermediate. Due to the characteristic of the chemically inert residues lining the active-site cavity conserved in CHSs but specifically substituted in other type III PKSs, they are thought to be critical to controlling the substrate and product specificity of the enzyme reaction (Morita et al., 2019).

The biosynthesis pathways of flavonoids have been relatively well elucidated in vascular plants (Winkel-Shirley Physiology BJP., 2001). However, the genes encoding the key enzymes involved in the biosynthesis of flavonoids and anthocyanins in ferns are largely unknown. Ferns are between bryophytes and seed plants in the evolution and produce flavones, chalcones, flavonols, and anthocyanins (Xie et al., 2015; Zhang et al., 2019). The fern species *Cyclosorus parasiticus* belongs to the family thelypteridaceae and is distributed throughout southern China. It is commonly known as a source of traditional Chinese medicine for its potential pharmacological activities (Butelli et al., 2008; Luceri et al., 2008). Three chalcone derivatives have been isolated from *C. parasiticus* (Wei et al., 2013). Recently, metabolomics integrated with transcriptomics has been widely used to investigate the biosynthesis of metabolites to reveal the biosynthetic pathways of metabolites in plants (Zhang et al., 2014; Dong et al., 2019; Sangpong et al., 2021). Therefore, it is feasible and useful to analyze the flavonoid biosynthesis pathway in the different developmental stages of *C. parasiticus* through the joint application of the two technologies. In our present investigation, integrated metabolome and transcriptome analysis indicated that some of the flavonoid biosynthesis-related genes were upregulated in S2, and the flavonoid metabolites content was higher in S2. Two CHSs, CpCHS1 and CpCHS2, were functionally characterized using recombinant proteins. The crystal structure of CpCHS1 was elucidated in the apo and presence of its final product naringenin, and detailed structural comparisons of CpCHS1 with EaCHS from *Equisetum arvense* and PpCHS from *Physcomitrella patens* revealed common differences between CHSs in the local conformation around the active site pocket (Liou et al., 2018). Mutation of several residues around the catalytic pocket of CpCHS1 dramatically reduced the *in vitro* catalytic activity of the enzyme, experimentally supporting their functional roles in substrate binding.

## Materials and Methods

### Plant Material and Sampling

**Figure 2:**
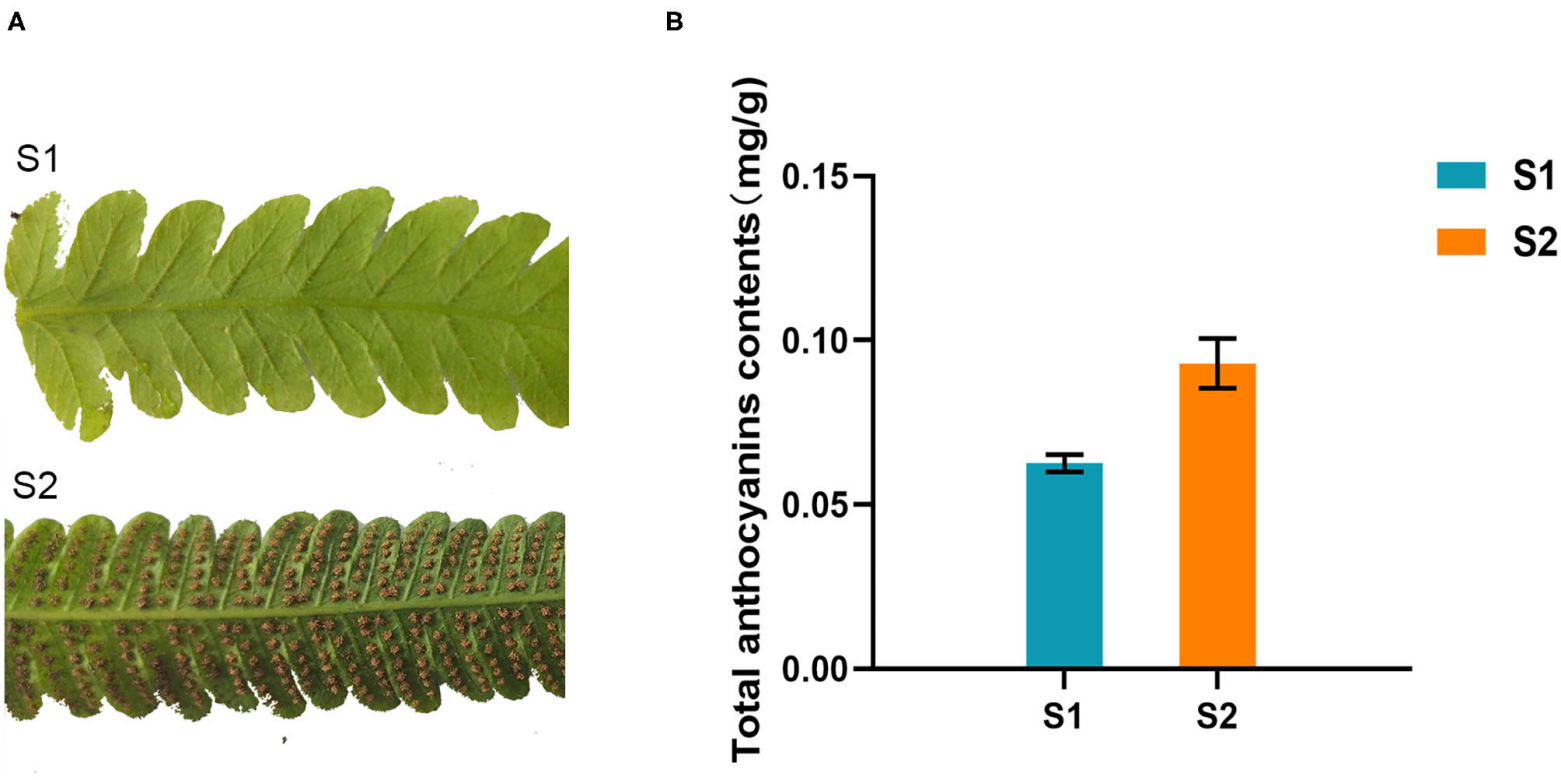
Total anthocyanins contents of different growing stages of *Cyclosorus parasiticus* fronds. **(A)** Different growing stages of *C. parasiticus* fronds. “S1” represents the first growing stage without spores; “S2” represents the second growing stage with brown spores. **(B)** Measurement of total anthocyanins in *C. parasiticus*. Results are means ± SD, *n* = 3.

Two different development stages fronds of the fern *C. parasiticus* were collected from the greenhouse of Shandong University, Shandong province, China. They were numbered “S1” (stage 1, the fronds without spores) and “S2” (stage 2, the fronds with brown spores) (Figure 2A). All the materials were frozen in liquid nitrogen immediately and then stored at −80°C until further analysis.

### Measurement of Total Anthocyanins Content

Anthocyanin analysis of two-stage fronds in *C. parasiticus* was carried out as the same way as previously reported (Zhang et al., 2013; Cheng et al., 2018).

### Sample Extraction and Metabolite Profiling Analysis

The freeze-dried fronds were grounded with zirconia beads by a mixer mill (MM 400, Retsch) at 30 Hz for 1.5 min. The powder (100 mg) was weighed and dissolved with 1.0 mL 70% methanol and extracted at 4°C overnight. After centrifugation at 10,000 g for 10 min, the sample extracts (supernatant) were collected and filtered by 0.22 μm pore size millipore filters, and then analyzed by an LC-MS/MS system (UPLC, Shim-pack UFLC SHIMADZU CBM30A, MS/MS Applied Biosystems 6500 QTRAP). Metabolites were identified by comparing the m/z values, the retention time (RT), and the fragmentation patterns with the standards in the database, and metabolites quantitate analysis was performed by MRM (multiple reaction monitoring) (Dong et al., 2019). QC samples were exported to analyze the sample repeatability under the same treatment. According to the result of OPLS-DA, the VIP (variable importance in the project) score was used to screen the significantly changed metabolites (SCMs). Differential metabolites with VIP ≥ 1, fold change ≥ 2, and fold change ≤ 0.5 were filtered as significantly changed metabolites. The MRM was carried out by Metware Biotechnology Co., Ltd. (Wuhan, China).

### RNA Extraction and cDNA Synthesis

Total RNA was extracted from frozen fronds, and three biological replicates were set for each sample. The isolated total RNA was removed, and residual DNA and the mRNA molecules were purified from total RNA using oligo (dT)-attached magnetic beads. After that, the enriched mRNA was fragmented and then reverse-transcribed with random hexamer-primers to produce the first-strand cDNA, followed by second-strand cDNA synthesis. The cDNA was carried out end-repair and 3′ adenylated, and then adapters were ligated to the modified cDNA fragments. The product was amplified by PCR, purified with Ampure XP Beads (AGENCOURT), and then heat denatured and circularized to form the final library. The prepared library was sequenced by BGISEQ-500, and 100 bp sequence reads were synthesized.

### RNA-Seq Data Analysis and Enrichment Analysis

SOAPnuke filter software was used for statistics and filtered by trimmomatic (Chen et al., 2018): the adaptor sequences (adaptor pollution), sequences with more than 5% unknown nucleotides (N), and low-quality sequence read (reads with a quality-value ≤ 10 account for more than 20% of the total base number) were removed from the data set. Clean reads were obtained to be mapped to the reference genome by Bowtie2, and RSEM software was used to calculate the expression levels of genes and transcripts. The edgeR package was adopted to identify differentially expressed genes (DEGs) of two samples. Fold Change ≥ 2 and adjusted *Q* ≤ 0.05 were filtered as the differentially expressed genes, and the DEGs were then mapped to GO functions enrichment analysis and Kyoto Encyclopedia of Genes and Genomes (KEGG) pathway analysis. The identified genes were annotated to the following databases: Nr (NCBI non-redundant protein sequences), Nt (NCBI non-redundant nucleotide sequences), Pfam (protein family), KOG/COG (clusters of orthologous groups of proteins), Swiss-Prot (a manually annotated and reviewed protein sequence database), KO (KEGG Ortholog database), and GO (gene ontology). The RNA-seq data of this research are available in the NCBI (accession No. PRJNA747631).

### Quantitative Real-Time Polymerase Chain Reaction (qRT-PCR)

Real-time PCR was carried out using Eppendorf realplex^2^ system. The reactions were performed by TB Green TM Premix Ex Taq TM (TliRnaseH Plus, Takara, China). An actin gene was set as an internal control, and three biological replicates were set for each sample. The program of qPCR analysis was set as follows: 95°C for 2 min, 40 cycles of 95°C for 15 s, 55°C for 15 s, and 68°C for 20 s. The sequence of actin and the primer sequence of the genes are listed in Supplementary Table 1. The 2^−ΔΔCT^ method was used for calculating the relative expression levels.

### Sequence Alignment, Phylogenetic Tree Analyses, and Homology Modeling

Two putative upregulated CHS-like DEGs were recognized from *C. parasiticus*, and the one selected by the screen was designated CpCHS1. In addition, one putative CHS gene, which is not the DEG, was recognized by searching the transcriptome database of *C. parasiticus* and named CpCHS2. Deduced CHS polypeptide sequences were aligned with those of other known plant CHSs using the Espript 3.0. A phylogenetic tree was constructed using MEGA 5.0 software, applying the neighbor-joining method (Tamura et al., 2011).

Homology models of CpCHS2 were generated using the modeling server SWISS-MODEL (https://swissmodel.expasy.org/), with the CpCHS1 structure serving as a template (Waterhouse et al., 2018).

### Gene Cloning, Heterologous Expression, and Protein Purification

The full-length sequences of *CpCHS1* (OK136250) and *CpCHS2* (OK136251) were separately amplified from the synthesized cDNAs with the primer pairs listed in Supplementary Table 2. The amplified ORFs were digested with corresponding restriction endonucleases, ligated into the *pET-32a* vector, and transformed into the *E. coli* BL21 (DE3). Protein expression and purification were performed using the methods previously reported (Ni et al., 2020).

For protein crystallization, *CpCHS1* was inserted into the *pETDuet-1* vector and transformed into *E. coli* Rosetta (DE3) strain and cultured in an LB liquid medium supplemented with ampicillin (50 μg ml^−1^) and chloramphenicol (25 μg ml^−1^) at 37°C in a shaker at 240 rpm. Protein purification was performed using the methods previously reported (Li et al., 2020). The purified protein was collected and concentrated for subsequent structural and biochemical studies.

Heterologous expression and purification of CpCHS1 mutants used the same procedure as described before (Li et al., 2020), and the primer pairs are listed in Supplementary Table 3.

### CpCHS Enzymatic Assay and Kinetic Analysis

A standard enzyme assay process and the preparation of substrates for enzyme activity (*p*-coumaroyl-CoA, caffeoyl-CoA, and cinnamoyl-CoA) were conducted following the methods previously reported by our lab (Ni et al., 2020). The enzyme assay mixture, including 50 μM starter CoA (*p*-coumaroyl-CoA, caffeoyl-CoA, and cinnamoyl-CoA), 100 μM malonyl-CoA, and 20 μg of the purified protein in 100 mM potassium phosphate buffer at pH 7.0 reached the final volume of 250 μl for 50 min. And the empty vector protein *pET-32a* was used as the negative control. For CpCHS2, the amount of recombinant proteins increased to 100 μg, and the reaction was extended to 3 h because the amount of protein expression was low. To determine the optimal pH, the enzymatic reactions were preceded at 35°C in various reaction buffers with pH values in the ranges of 5.0–6.5 (2-morpholino-ethanesulfonic acid, an MES buffer), 6.5–7.5 (a Tris-HCl buffer), and 7.5–9.0 (a K_2_HPO_4_-KH_2_PO_4_ buffer). To test the optimal reaction temperature, the reactions were incubated at pH7.5 (K_2_HPO_4_-KH_2_PO_4_ buffer) for 50 min at different temperatures (25–65°C). To determine the kinetic parameters of CpCHS1 for *p*-coumaroyl-CoA or cafferoyl-CoA, an enzymatic assay containing a 100 mM K_2_HPO_4_-KH_2_PO_4_ buffer (pH 7.5), 2 μg of purified recombinant enzyme CpCHS1, 300 μM of malonyl-CoA, and varying concentrations (1, 3, 5, 10, 15, 25, 50, and 75 μM) of *p*-coumaroyl-CoA or caffeoyl-CoA were performed at 45°C for 10 min in a final volume of 100 μl. For the determination of kinetic parameters for malonyl-CoA with CpCHS1, its concentration varied from 5 to 150 μM (5, 10, 20, 40, 50, 75, 100, and 150 μM), and the *p*-coumaroyl-CoA was fixed in the 400 μM. The reactions terminated by the addition of 10 μl of 10% glacial acetic acid, and the extraction and the analysis were performed using the method previously reported (Ni et al., 2020). The kinetic parameters were calculated from Lineweaver-Burk plots and analyzed with Graphpad Prism 5 software.

### Crystallization, Data Collection, and Structure Determination

To obtain the crystals of CpCHS1 in complex with substrates, the protein was incubated with 5 mM substrates on ice for 60 min before crystallization. Crystals for CpCHS1 and CpCHS1- substrates complex were grown at 20°C for 7 days using the sitting-drop vapor diffusion method by mixing 0.5 μl of protein with 0.5 μl of reservoir solution containing 0.2 M sodium chloride, 0.1 M Tris-HCl, 20% w/v PEG 4000, and pH 8.0. Crystals for data collection were directly flash-frozen in a nitrogen stream at 100 K. The data of CpCHS1 in apo form and substrates-binding forms were collected at Shanghai Synchrotron Radiation Facility (SSRF) beamline BL19U1/18U1/17U1 and were processed using the HKL3000 package (Minor et al., 2006). Structures of the CpCHS1 and the CpCHS1-substrates complex were solved by Molecular Replacement using PHENIX (Adams et al., 2010) with 6DXB structure as the initial model. All the models were refined with PHENIX and manually built with Coot (Emsley and Cowtan, 2004). The data collection and refinement statistics are summarized in Supplementary Table 4.

### Data Availability

The atomic coordinates and structure factors have been deposited in the Protein Data Bank with accession codes 7VEY, 7VEZ, and 7VF0.

## Results

### Measurement of Total Anthocyanins in Fronds of *C. parasiticus*

The total anthocyanins content of two kinds of fronds (one with brown spores was named S2, and the other without spores was named S1) of *C. parasiticus* was measured. The results showed that the total anthocyanins content of S1 was about 0.063 mg/g of fresh weight, which was lower than the 0.093 mg/g fresh weight of S2 (Figure 2B). It revealed that the content of anthocyanins changed during the two stages in *C. parasiticus* fronds, and the green fronds with brown spores were detected more anthocyanins.

### Metabolic Analysis of Two Kinds of *C. parasiticus* Fronds

**Figure 3:**
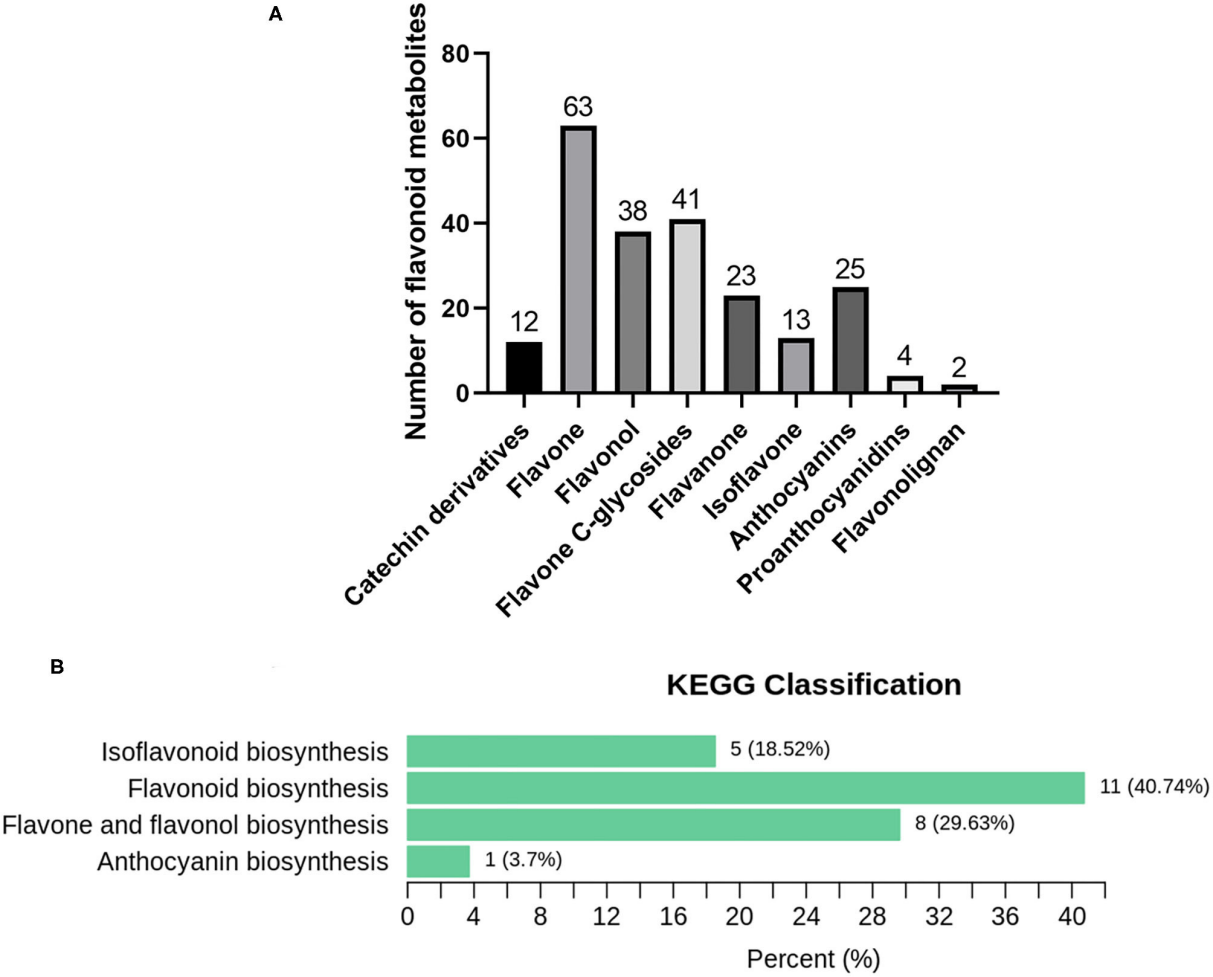
Metabolomics analysis results. **(A)** Flavonoid metabolites in *C. parasiticus*. **(B)** The differential metabolites Kyoto Encyclopedia of Genes and Genomes (KEGG) classification of the fronds of *C. parasiticus*. The proportion and the number of metabolites are marked in the figure.

The flavonoid metabolites of *C. parasiticus* fronds without or with brown spores were investigated based on LC-MS/MS analysis. A total of 221 metabolites were identified and conducted qualitative and quantitative analysis, including 63 flavones, 25 anthocyanins, 38 flavonols, 23 flavanones, 13 isoflavones, four proanthocyanidins, two flavonolignans, 41 flavone C-glycosides, and 12 catechin derivatives (Figure 3A). According to the combined metabolites fold change analysis with OPLS-DA model analysis, totally 69 markedly changed metabolites were screened in the flavonoid pathway with screening criteria: fold change ≥ 2 or fold change ≤ 0.5 and VIP ≥1. Of all the markedly changed metabolites, we discovered that the eight categories were changed and chose 12 upregulated metabolites and four downregulated metabolites as representative compounds (Supplementary Figure 1). In total, 10 anthocyanins were identified in fronds of *C. parasiticus*, only the Peonidin *O*-malonylhexoside was significantly lower in S1 vs. S2. The differential flavonoid metabolites from each comparison group were screened by using the KEGG database. According to the KEGG annotation result, all of the markedly changed metabolites were constructed into KEGG classification (Figure 3B); it indicated that the difference mainly focused on the flavonoid and anthocyanin biosynthesis. It supports that the changes between sample fronds were due to the flavonoid and anthocyanin abundant difference.

### Transcriptome Analysis of *C. parasiticus* Fronds at Two Different Developmental Stages

**Figure 4:**
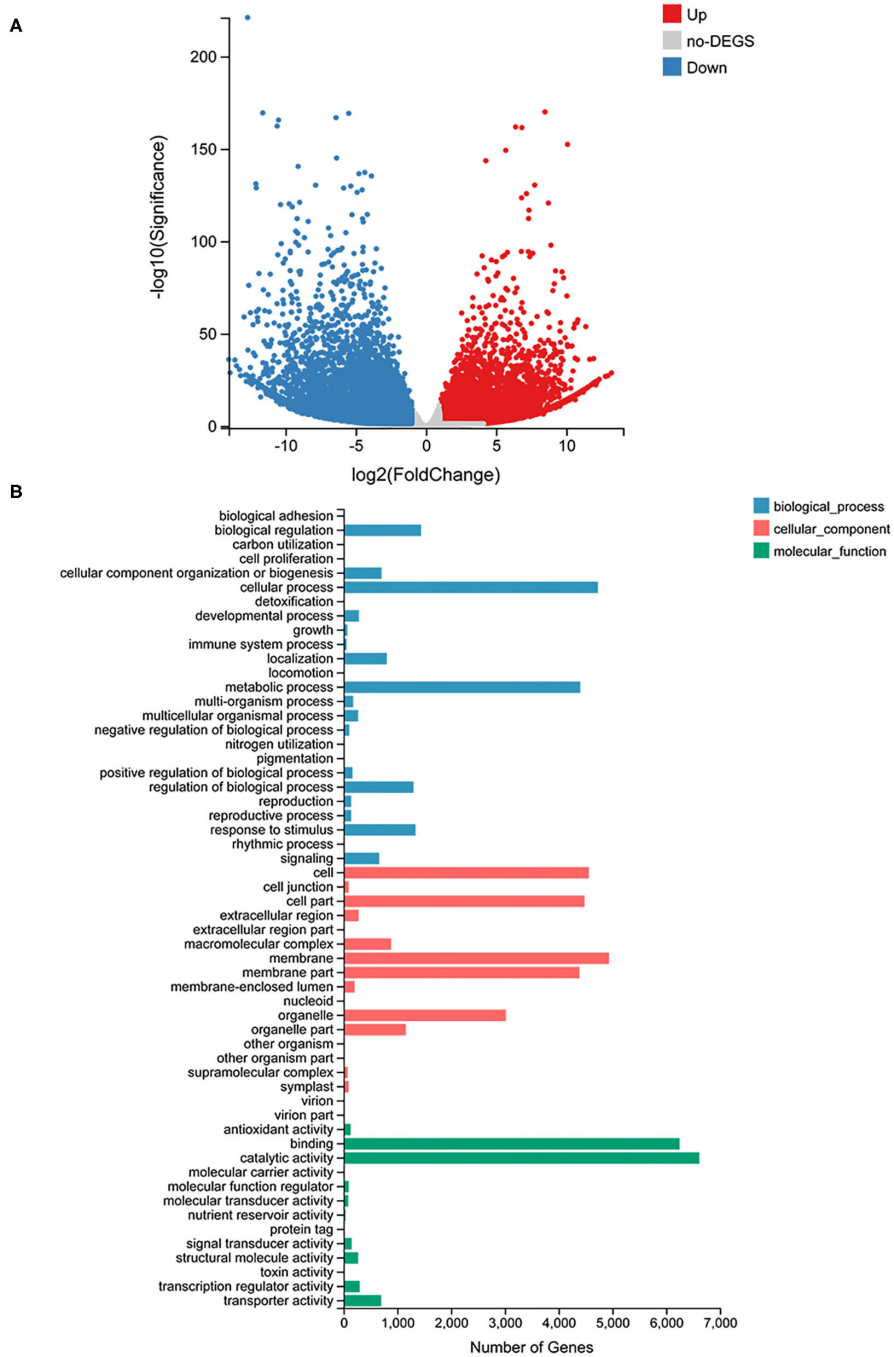
RNA-seq analysis results. **(A)** The volcano plot of all genes detected in *C. parasiticus* fronds. The red dots represent upregulated genes, the blue dots represent downregulated genes, and the gray dots mean no difference between S1 and S2. **(B)** Go classification of differently expressed genes detected in *C. parasiticus* fronds. The red represents the cellular part, the blue represents the biological process part, and the green means the molecular function part.

To identify genes differentially expressed in the fronds of S1 compared with those of S2, the transcriptomes of the fronds were sequenced. According to the statistics, 76,064 unigenes were obtained, and all the assembled unigene sequences were further targeted for BLASTx analysis for functional annotation. Gene expression profiling was screened based on fold change ≥ 2 and Q ≤ 0.05; a total of 26,188 different expresses genes (DEGs) were identified and measured, among which 12,319 genes were upregulated and 13,869 genes were downregulated in the S2 compared with S1. The result was shown on the volcano plot (Figure 4A). The GO enrichment analysis of DEGs was divided into three parts: biological process, cellular component, and molecular function category (Figure 4B). The result showed that the cellular process is the most abundant among 25 groups of the biological process, followed by the metabolic process. In the cellular component, the top three classified groups are the membrane, cell, and cell part among 18 groups. And under the molecular function process, catalytic activity and binding are the most abundant groups compared with other groups. The GO annotation results revealed that unigenes of *C. parasiticus* encode diverse metabolism-related proteins.

In addition, the KEGG pathway systematic analysis was conducted to find enriched metabolic pathways and functions of gene products. The top 20 terms with the minimum Q-value were chosen to mapping KEGG pathways enrichment; the result revealed that the most enriched pathway was metabolism-related, and the flavonoid biosynthesis pathway showed significantly enriched (Supplementary Figure 2). The result showed that the most differently expressed genes were related to metabolism, and there were significant differences in the content of flavonoids and the expression of related genes between S1 and S2 fronds.

### Analysis of Differentially Expressed Genes Involved in Flavonoids and Anthocyanins Biosynthesis Pathways

Metabolomic data indicate that the flavonoid (catalyzed by CHS, F3H, and F3'H) and anthocyanin (catalyzed by ANS) content was higher in S2 vs. S1; to understand this finding, transcriptomic data were analyzed, and a large number of differentially expressed genes were detected in fronds of *C. parasiticus*. Among them, unigenes involved in flavonoids, especially the anthocyanin biosynthesis pathway, were further screened by exploiting the key genes in the metabolism of two different development stages fronds in *C. parasiticus*.

**Figure 5:**
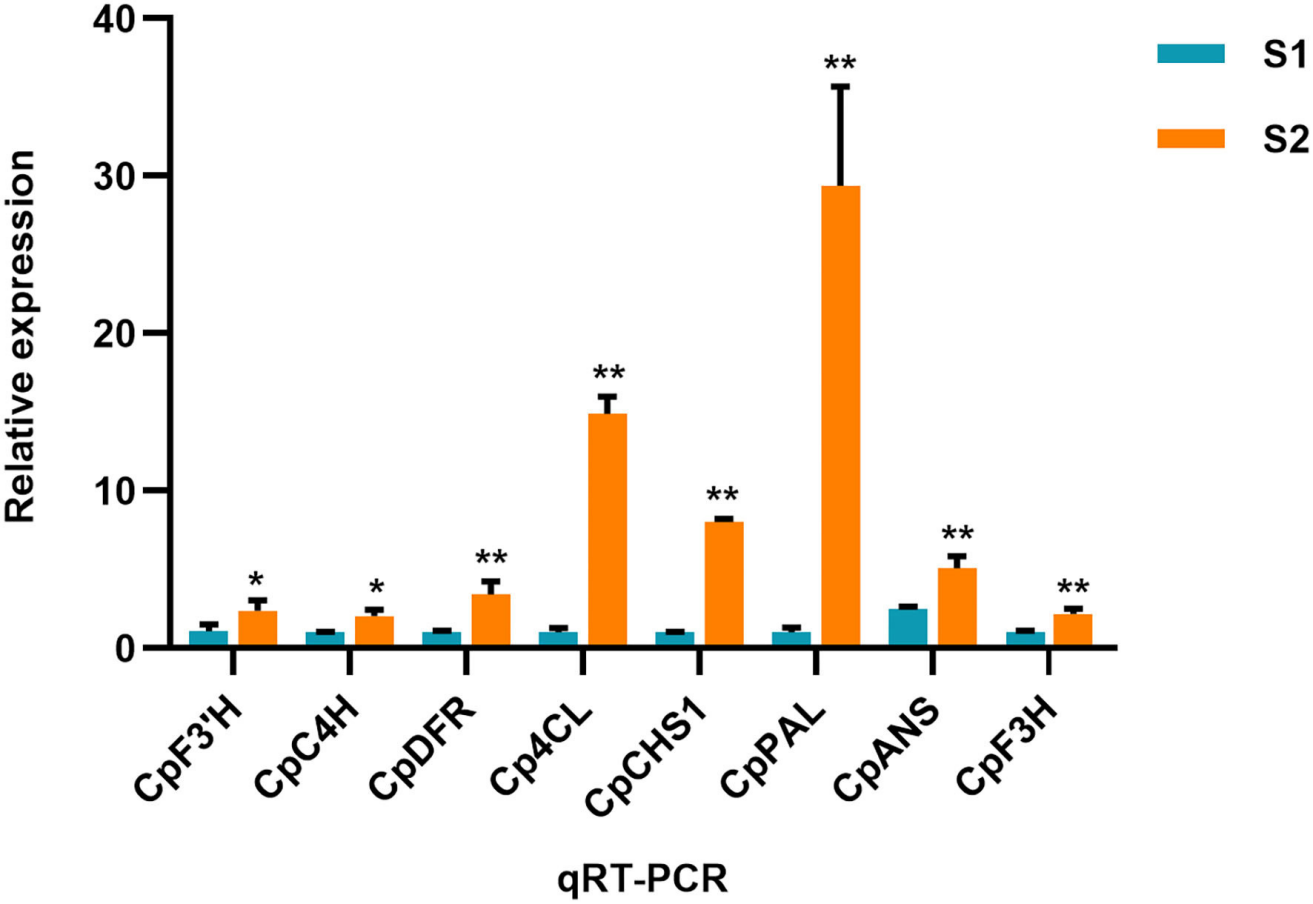
qRT-PCR result of the flavonoid biosynthesis-related genes. The genes include CL1149.Contig3_All (annotated as F3'H), Unigene11283_All (annotated as C4H), CL871.Contig7_All (annotated as DFR), CL1096.Contig5_All (annotated as 4CL), CL5031.Contig1_All (annotated as CHS), CL5392.Contig1_All (annotated as PAL). ^*^PCR res *t* < 0.05. ^**^means *t* < 0.01; results are means ± SD, *n* = 3.

The number of up/downregulated genes of the flavonoid biosynthesis pathway is shown in Table 1. To further validate the result of the transcriptome, the expression level of several related genes was examined by qRT-PCR (Figure 5). The result showed that PAL (phenylalanine ammonia lyase), C4H (cinnamate 4-hydroxylase), 4CL (4-coumarate coenzyme A ligase), CHS, F3′H, DFR, and ANS were all upregulated in S2 compared with S1, implying these structure genes were essential for *C. parasiticus* flavonoid biosynthesis.

**Table 1:** Candidate genes related to the biosynthesis of flavonoids of *Cyclosorus parasiticus*.

**Function**	**Gene**	**Enzyme**	**Ko id**	**No. up**	**No. down**	**No. all**
Flavone and flavonol biosynthesis	CHS	Chalcone synthase	K00660 (2.1.3.74)	2	4	6
	CHI	Chalcone isomerase	K01859 (5.5.1.6)	0	4	4
	F3H	Flavanone 3-hydroxylase	K00475 (1.14.11.9)	3	0	3
	F3'H	Flavonoid 3′-hydroxylase	K05280 (1.14.13.21)	6	0	6
	F3'5'H	Flavonoid 3′,5′-hydroxylase	K13083 (1.14.13.88)	1	1	2
	FLS	Flavonol synthase	K05278 (1.14.11.23)	1	5	6
Anthocyanin biosynthesis	DFR	Dihydroflavonol 4-reductase	K13082 (1.1.1.219)	5	8	13
	ANS	Anthocyanidin synthase	K05277 (1.14.11.19)	3	2	5

*No. all, the total number of DEGs mapped to the Kyoto Encyclopedia of Genes and Genomes (KEGG) pathway.*

*o. up, the number of uni-transcripts with expression significantly upregulated in the S2 development stage of C. parasiticus compared with S2 stages.*

*No. down, the number of uni-transcripts with expression significantly downregulated in the S2 development stage of C. parasiticus compared with S2 stages*.

### Identification of CHS Genes in *C. parasiticus*

Two putative CHSs (CL5031.Contig1 and Unigene4665) were recognized from the transcriptomic data of *C. parasiticus*. CL5031. Contig1 was upregulated in S2, and Unigene4665 showed a low-expression level that can hardly see the difference between S1 and S2. CL5031.Contig1 and Unigene4665 were named *CpCHS1* and *CpCHS2* for further functional characterization.

To elucidate the evolutionary relationship between CpCHSs isoforms and CHSs from other plant species, we performed a phylogenetic analysis by comparing the amino acid sequences of the two CpCHSs, along with previously characterized CHS proteins from other plant species (Supplementary Figure 3). The result revealed that the CpCHSs clustered into the pteridophyta family and had a close relationship with the LoCHS1 (*L. orbiculata*, QDF63003.1) (Ni et al., 2020) and EaCHS (*E. arvense*, Q9MBB1.1).

### Characterization and Function Analysis of CHS in *C. parasiticus*

**Figure 6:**
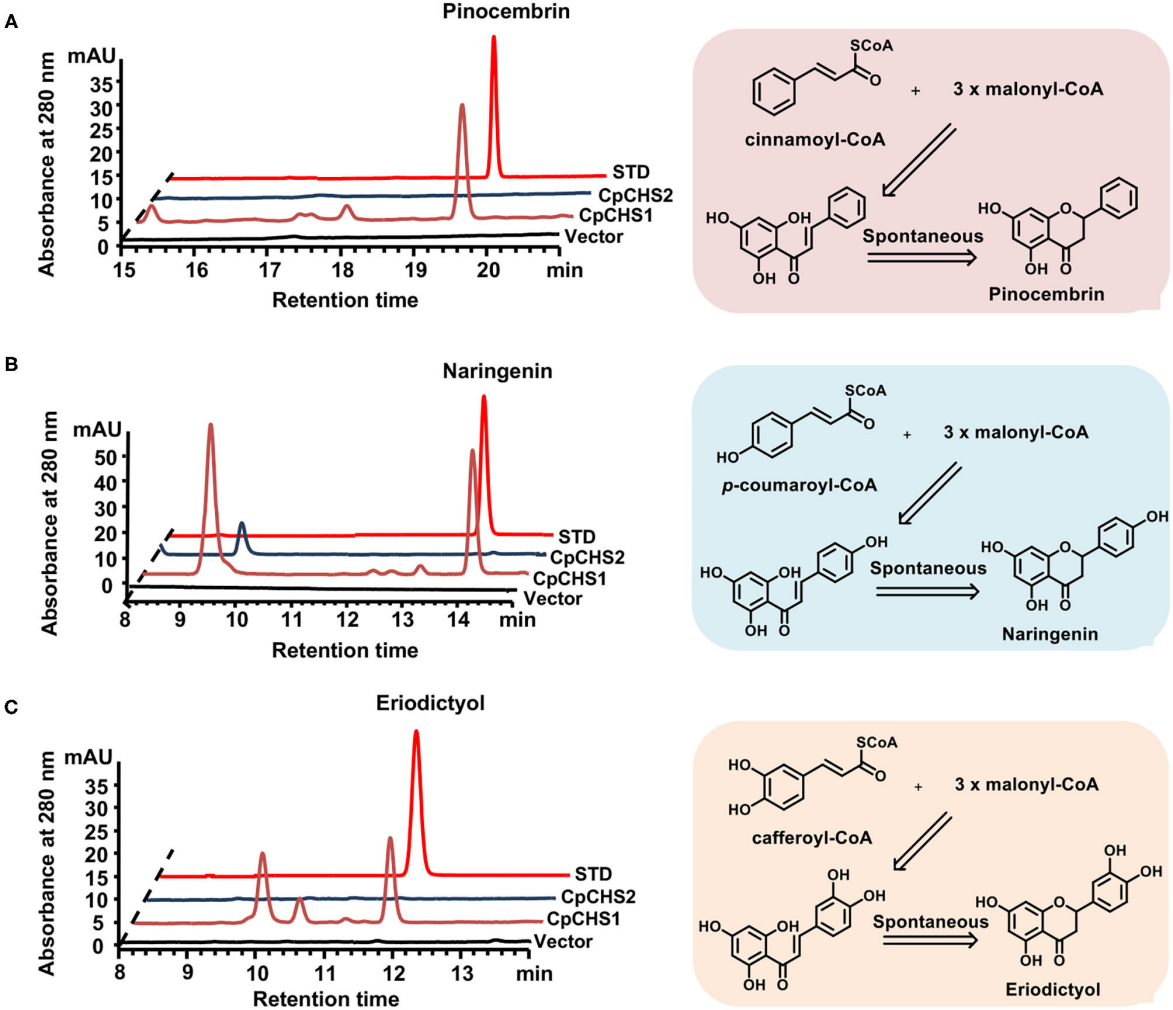
*In vitro* assays of recombinant CpCHS1 and CpCHS2. **(A)** HPLC profiles of the products generated by the empty vector control and CpCHS1, CpCHS2 using cinnamoyl-CoA as a substrate. **(B)** HPLC profiles of the products generated by the empty vector control and CpCHS1, CpCHS2 using *p*-coumaroyl-CoA as a substrate. **(C)** HPLC profiles of the products generated by the empty vector control and CpCHS1, CpCHS2 using caffeoyl-CoA as a substrate. STD, standard; vector: *pET-32a*.

To demonstrate functional activity of CpCHSs, recombinant proteins were expressed in *E. coli* and purified (Supplementary Figure 4) the enzyme activity assay by using *p*-coumaroyl-CoA, caffeoyl-CoA, and cinnamoyl-CoA as substrates. The reaction products of CpCHSs were analyzed by HPLC in comparison to authentic standards (Figure 6), and the results indicated that CpCHS1 could catalyze the biosynthesis of pinocembrin, naringenin, and eriodictyol with the cinnamoyl-CoA, *p*-coumaroyl-CoA, and caffeoyl-CoA as substrates, respectively. However, CpCHS2 was inactive toward all three substrates (Figure 6).

We detected the catalytic activity of CpCHS1 at different temperatures and pH using *p*-coumaryol-CoA as a substrate; the result revealed that the optimal temperature for CpCHS1 was 45°C, and the optimum pH was 7.5 (Supplementary Figure 5). Under optimum pH and temperature, the CpCHS1 recombinant enzyme showed an apparent *k*_*cat*_/*K*_*m*_ of 1.47 × 10^5^ M^−1^min^−1^ for *p*-coumaryol-CoA. The catalytic efficiency of CpCHS1 for caffeoyl-CoA was lower than that of *p*-coumaryol-CoA by comparing the *k*_*cat*_*/K*_*m*_ value (1.02 × 10^4^M^−1^ min^−1^ for caffeoyl-CoA). CpCHS1 showed *k*_*cat*_/*K*_*m*_ of 3.58 × 10^4^ for malonyl-CoA when a fixed concentration of 400 μM *p*-Coumaroyl-CoA was used as a substrate (Table 2).

**Table 2:** Steady-state kinetic parameters of CpCHS1.

**Substrate**	**Protein**	***K_m_*** **(μM)**	***V_max_*** **(nmol mg^−1^ min^−1^)**	**K_cat_ (min^−1^)**	***K_cat_/K_m_*** **(M^−1^min^−1^)**
Malonyl-CoA^a^	CpCHS1	32.39 ± 5.03	17.93 ± 0.97	1.16 ± 0.06	3.58 × 10^4^
*p*-Coumaroyl-CoA^b^	CpCHS1	7.20 ± 1.07	16.31 ± 0.69	1.06 ± 0.04	1.47 × 10^5^
Caffeoyl-CoA^b^	CpCHS1	13.35 ± 1.94	2.12 ± 0.11	0.14 ± 0.01	1.02 × 10^4^

a *A fix concentration of 400 μM p-Coumaroyl-CoA was used as a substrate.*

b *A fix concentration of 300 μM malonyl-CoA was used as a substrate*.

### The Overall Structure of the CpCHS1

**Figure 7:**
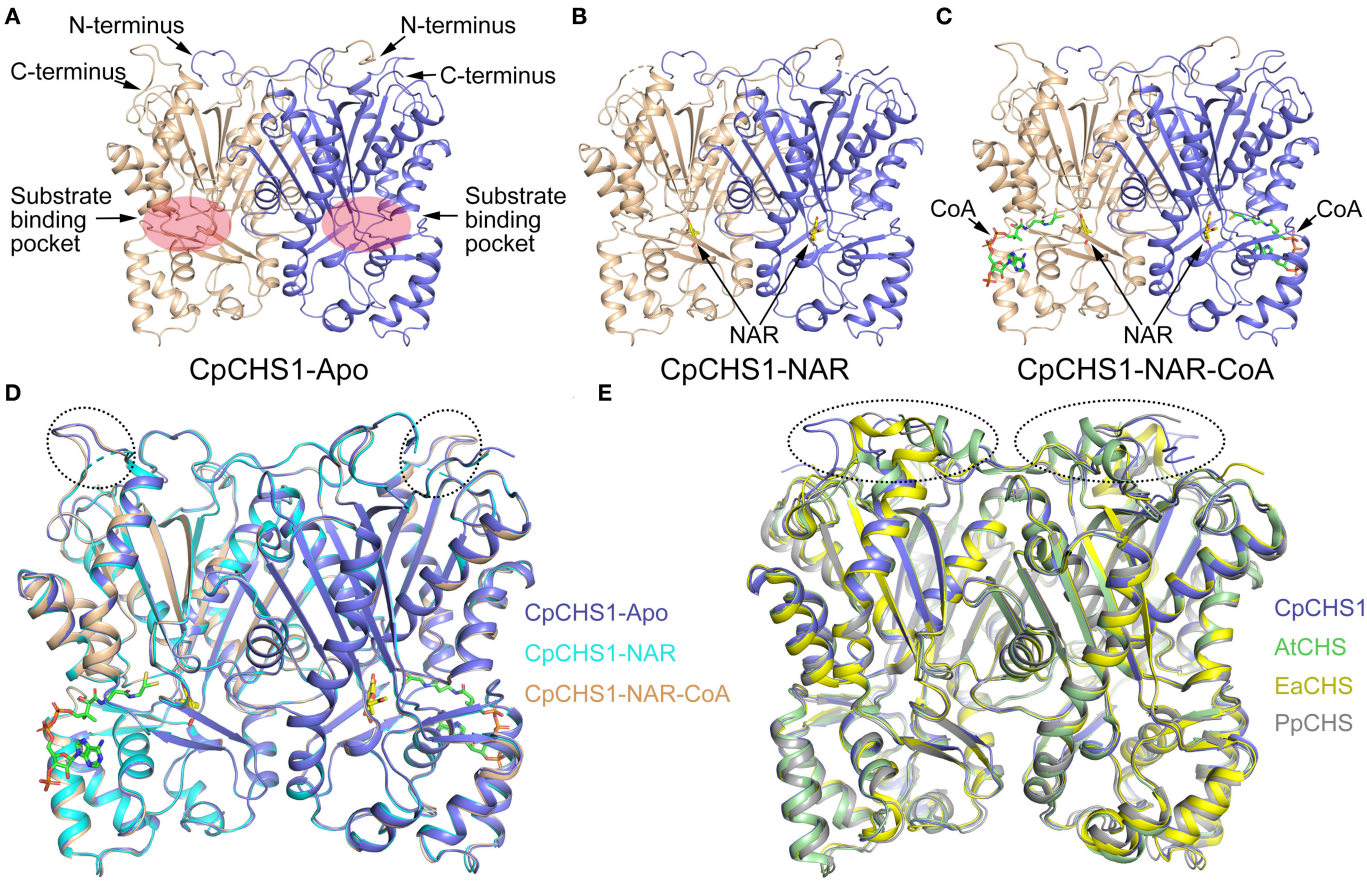
Crystal structure analysis of CpCHS1. **(A)** Apo structure of CpCHS1. **(B)** CpCHS1 structure complex with naringenin. **(C)** CpCHS1 structure complex with naringenin and CoA. **(D)** Structure comparison analysis of CpCHS1-Apo, CpCHS1- NAR, and CpCHS1-NAR-CoA. **(E)** Structure comparison analysis of CpCHS1-Apo, AtCHS (PDB: 6DXB), EaCHS (PDB: 6DX9), and PpCHS (PDB: 6DX7).

To understand the molecular basis of *C. parasiticus* CHS1, the crystal structure of CpCHS1 in apo form was determined at 1.9 Å using the molecular replacement method. The structure contains the full-length CpCHS1, except that residues 1–5 and 402–404 are disordered (hereafter CpCHS1-Apo, Figure 7A). Similar to previously reported crystal structures of plant chalcone synthase, we found that the final model of CpCHS1 forms symmetric homodimers, and it was comprised of the same α*βαβα* thiolase fold (Ferrer et al., 1999; Liou et al., 2018). Attempting to study the mechanism of substrate binding and catalysis of CpCHS1, we made numerous efforts aiming to determine the structure of a substrate-enzyme complex. In the end, we solved the crystal structure of CpCHS1 in complex with the reaction product naringenin (hereafter CpCHS1-NAR), and naringenin and CoA (hereafter CpCHS1-NAR-CoA), and determined their structures to 2.4 Å (Figures 7B,C; Supplementary Table 4). The naringenin and CoA bury in the substrate binding pocket, which is formed by the upper and lower domains of CpCHS1, and their electron densities are visible in the complex structures (Supplementary Figure 6). Compared with CpCHS1-Apo, we found the loop, containing residues 299–303, is disordered in the structure of CpCHS1-NAR, but the overall structures of CpCHS1-NAR and CpCHS1-NAR-CoA have very few conformational changes, with the root-mean-square deviation (RMSD) values of 0.234 and 0.187 Å, respectively (Figure 7D).

To comprehend the structural differences for the evolution of CHS across some major land plant lineages, the angiosperm *Arabidopsis thaliana* CHS (AtCHS, PDB ID code 6DXB), the monilophyte *E. arvense* CHS (EaCHS, PDB ID code 6DX9), and the bryophyte *P. patens* CHS (PpCHS, PDB ID code 6DX7) are chosen and compared their structures with CpCHS1 (Figure 7E), whose RMSD are 0.314, 0.315, and 0.666 Å, respectively. The results showed that their structures were very similar, and the biggest differences were located in the loop regions on the top of the upper domain and further confirmed by the amino acid sequence (Supplementary Figure 7). We also analyzed the catalytic triad in the substrate-binding pocket and found the key catalytic residue Cys170 is oxidized to sulfenic acid like other higher plants (Liou et al., 2018) (Supplementary Figure 6). These data suggested that CpCHS1 showed a highly similar conformation and shared a similar general catalytic mechanism to another plant CHS.

### The Substrate-Binding Pocket of CpCHS1

**Figure 8:**
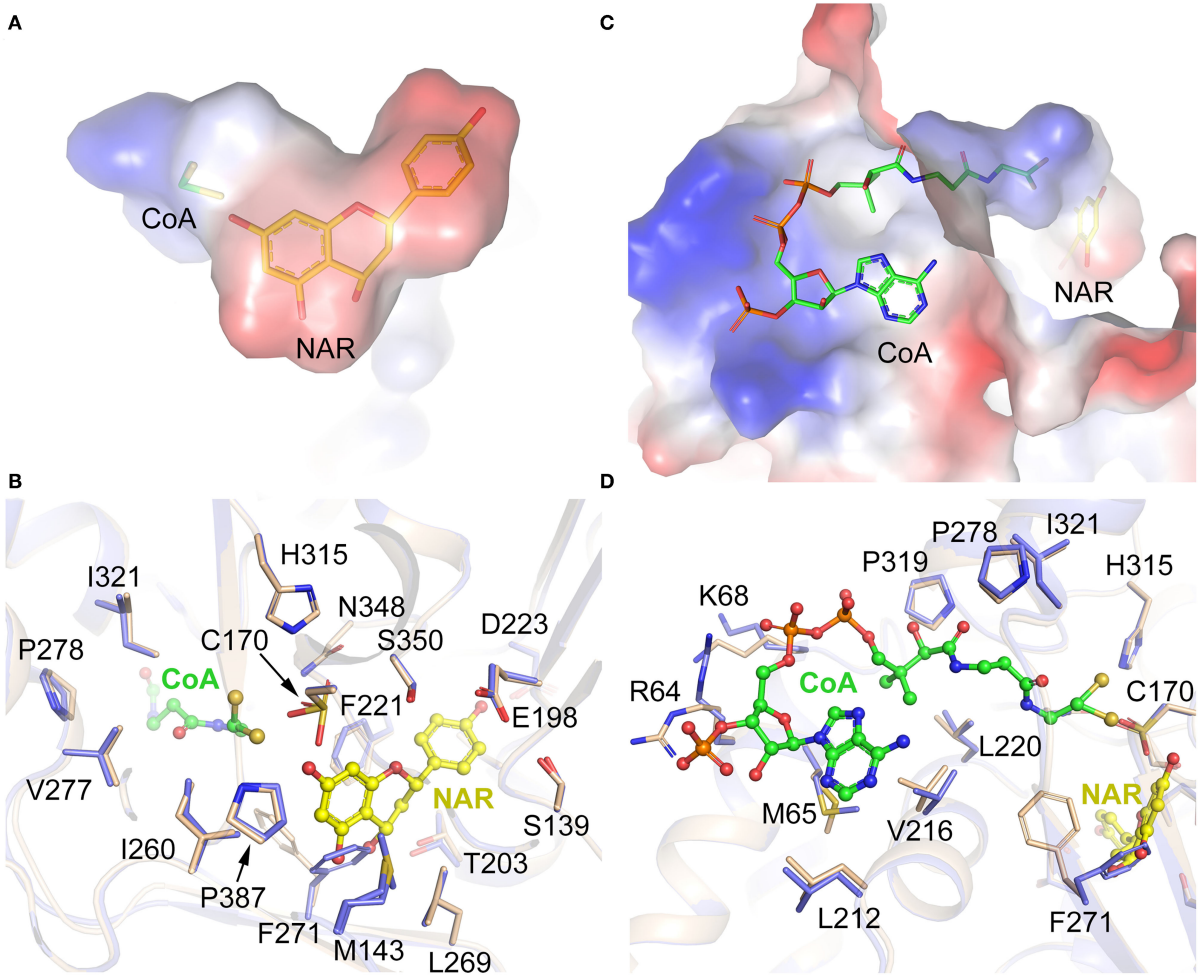
Substrate-binding pocket analysis of CpCHS1. **(A)** The binding pocket of naringenin. **(B)** Interaction of amino acid residues with naringenin in the binding site. **(C)** The binding pocket of CoA. **(D)** Interaction of amino acid residues with CoA in the binding site.

The CpCHS1-NAR and CpCHS1-NAR-CoA structures clearly show the specific binding pocket of naringenin and CoA. And the pocket, which is mainly formed by hydrophobic residues, could be divided into two parts: a short “L” shape space for naringenin and a long “L” shape space for CoA (Figures 8A,C). Besides the catalytic triad residues Cys170, His315, and Asn348, the residues of Ser139, Glu198, Thr203, Phe221, Asp223, Ile260, Leu269, Phe271, Ser350, Pro387, and together with Met143 from the adjacent monomer to constitute the naringenin-binding pocket (Figures 8A,B). The CoA-binding pocket is mainly formed by residues Arg64, Met65, Lys68, Leu212, Val216, Leu220, Pro278, Pro319, and Ile321, and CoA penetrates the enzyme-active site through a long CoA-binding tunnel (Figures 8C,D). To comprehend the conformational changes induced by naringenin and CoA binding, the detailed structures in the substrate-binding pocket of CpCHS1-Apo and CpCHS1-NAR-CoA were compared and analyzed. We found that the residue Phe271 occupies the substrate or product-binding position in the state of unbound naringenin, and this may represent the inactive state of CpCHS1 (Figure 8B). When CoA binds in the substrate-binding pocket of CpCHS1, the conformational change has occurred in the side chain of residues Arg64 and Lys68, which form hydrogen bonds with phosphate moieties of CoA (Figure 8D).

To study the conservation of critical amino acid residues in the substrate-binding pocket, we compared CpCHS1 with the chalcone synthase of *M. sativa* (1CGK, the first reported structure of chalcone synthase). CpCHS1 has a very similar conformation with MsCHS in the overall structure (RMSD of 0.357 Å) (Supplementary Figure 8A). And the amino acid residues that form the substrate-binding pocket are also identical, except for Leu199/Val193 (Supplementary Figure 8B). Meanwhile, the sequence comparative analysis of CpCHS1 with other plant chalcone synthases also revealed that the amino acid residues that form the substrate-binding pocket are very similar (Supplementary Figure 7). So, the results further suggested that the plant chalcone synthases are highly conserved in evolution.

**Figure 9:**
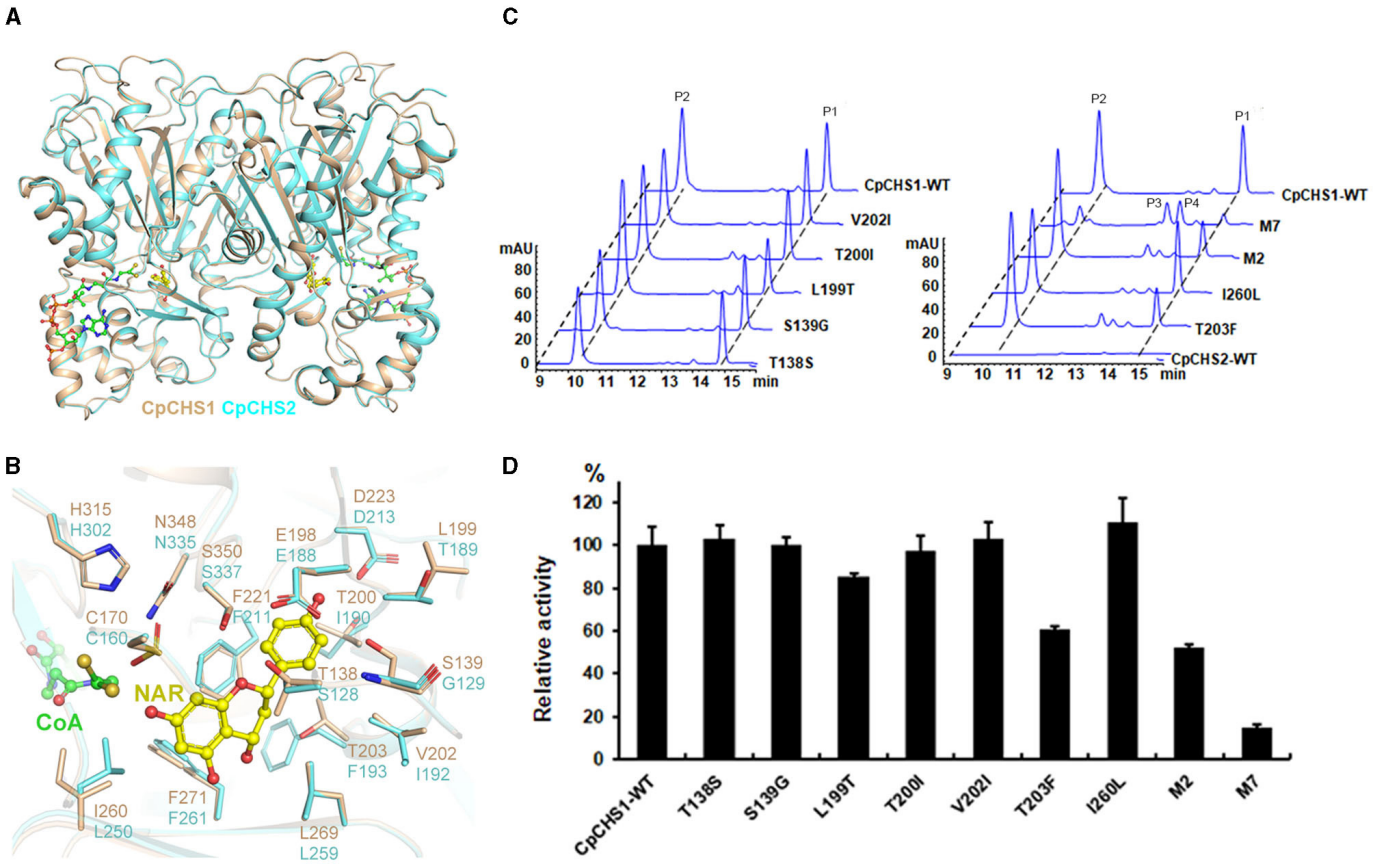
Structure comparison and mutation analysis of CpCHS1 and CpCHS2. **(A)** Comparison of the cartoon models of the CpCHS1 and CpCHS2 homology model. **(B)** Comparison of the models of CpCHS1 (brown) and CpCHS2 (cyan), The different amino acids in the catalytic pockets of CpCHS1 and CpCHS2 are highlighted. **(C)** HPLC profiles of the products generated by the CpCHS1 wild type and the CpCHS1 mutant using *p*-coumaroyl-CoA as a substrate, P1: naringenin, P2: CTAL, P3, P4: BNY, or its isomer. **(D)** Influence of the substrate-binding pocket of residue mutations on CpCHS1 activity; results are means ± SD, *n* = 3.

Our enzymatic assay indicated that the translated protein of CpCHS2 is an inactivated enzyme, although *CpCHS2* exhibited a high nucleotide sequence identity (68.4%) with *CpCHS1*. To explore why CpCHS2 has no catalytic function, the homology model of CpCHS2 was generated using the modeling server SWISS-MODEL with the CpCHS1 structure serving as a template. We found that CpCHS2 has a very similar conformation to CpCHS1 (RMSD of 0.186 Å) (Figure 9A). The amino acid residues that form the substrate-binding pocket were further analyzed, and most of them are the same, except for Thr138/Ser128, Leu199/Thr189, Thr200/Ile190, Thr203/Phe193, Val202/Ile192, Ile260/Leu250, and Ser139/Gly129 in CpCHS1 and CpCHS2, respectively (Figure 9B). So, the corresponding CpCHS1 mutants (with the replacement of the corresponding amino acid in CpCHS2) were constructed and expressed for enzymatic activity analysis. The results show that the mutation T203F significantly decreased the enzymatic activity, whereas the mutations I206L, T138S, S139G, T200I, V202I, and L199T mildly to moderately affect the enzymatic activity. The M2 mutant (L199T and T203F double mutations) was generated, and its enzymatic activity has a significant reduction comparing with the wild type of CpCHS1 (Figures 9C,D). Furthermore, the M7 mutant, including the aforementioned seven mutations, had a significant decrease in enzymatic activity. There were two minor peaks that clearly appeared in HPLC charts in the case of the mutants. According to the LC-MS results and the reaction mechanism, it indicated that P1 was naringenin, and P2 showed the same molecular weight with coumaroyltriacetic acid lactone (CTAL) (Figure 9C; Supplementary Figure 9). The two minor peaks showed the same molecular weight with bisnoryangonin (BNY), indicating they probably were BNY or its isomer. However, it could not be identified due to the minor amounts (Figure 9C; Supplementary Figure 9). So, these data suggested that the seven amino acid residues, which constitute the substrate-binding pocket, play an extremely important role in the activity and status of chalcone synthase. And these results imply CpCHS2 maybe has no function *in vivo*.

## Discussion

The combination of transcriptome and metabolome is an important field in multi-omics research. This method is used to discover the molecular mechanism of plant phenomena (Dobrowolska et al., 2017; Jiang et al., 2020). The primary goal of this study was to explore the mechanisms responsible for the accumulation of flavonoids and the genes related to their biosynthesis in *C. parasiticus* fronds. For this purpose, we analyzed the two kinds of fronds from different development stages of the fern *C. parasiticus*. Correlated gene information in the transcriptome with phenotypic information in the metabolome, a total of 26,188 differentially expressed genes and 69 different metabolites were identified. Most components of flavonoids showed drastically higher abundances in S2 than those in S1. In this study, we detected a significant accumulation of 10 types of anthocyanins, among which the most abundant one was the peonidin *O*-hexoside in *C. parasiticus*. In plants, anthocyanins and their derivatives are produced through the flavonoids biosynthesis pathway. In an analysis of the flavonoids biosynthesis pathway, we identified 56 unigenes-encoding eight enzymes that are involved in flavonoids biosynthesis in fronds of *C. parasiticus*. The qRT-PCR assays confirmed the reliability of RNA-seq analysis, and the identified genes are involved in flavonoid biosynthesis as candidate genes.

Chalcone synthase is the first step in the flavonoid pathway of plants. The functional CHS in plants has been widely studied, and the critical amino acid has been identified, such as the MpCHS (*Marchantia paleacea*), EaCHS (*E. arvense*), and AtCHS (*A. thaliana*) (Liou et al., 2018; Yu et al., 2018). In this study, we cloned two CHS in the fern *C. parasiticus*. Sequence analysis revealed that the CpCHS1 shares characteristics with many other CHSs and possessed the conserved active site, but the CpCHS2 was not (Supplementary Figure 7). The function analysis indicated that the CpCHS1 showed broad catalytic activities under *in vitro* enzyme assay; in contrast, CpCHS2 showed no obvious catalytic activities under the same reaction conditions. The results indicate that the differently expressed gene CpCHS1 was the flux-limiting gene leading to the anthocyanin and flavonoids accumulation in S2. The reaction kinetics of CpCHS1 was detected and combined with MpCHS (*M. paleacea* KY968327), PaCHS (*Plagiochasma appendiculatum*, AHY39238), and MsCHS (*M. sativa*, AAB41559.1) *k*_*cat*_/*K*_*m*_ values (Jez et al., 2000; Yu et al., 2015); the CpCHS1 toward *p*-coumaryol-CoA was 1.47 × 10^5^ M^−1^ min^−1^, a value which exceeded that shown by MpCHS (3.08 × 10^4^ M^−1^ min^−1^) and PaCHS (6.62 × 10^4^ M^−1^ min^−1^). However, the MsCHS (8.42 × 10^5^ M^−1^ min^−1^) displays a six-fold preference for *p*-coumaryol-CoA compared to CpCHS1. Overall, CpCHS1 showed higher catalytic efficiency than those associated with their bryophyte counterparts but lower than that of angiosperm counterparts.

The structures of CHS alone and complexed with a series of product analogs provide a framework for understanding the biosynthesis of plant polyketides like chalcone and naringenin. The overall structure of CpCHS1 displayed as a homodimer in solution contains a conserved Cys-His-Asn catalytic triad and exists as an α*βαβα* pseudo symmetric motif (Figure 7A). When viewed in surface representation, it contains two functionally independent active sites; bound CoA thioesters and product analogs occupy both active sites of the homodimer. In the CpCHS1 complex structures, there is a CoA-binding pocket, which is described as a long “L” shape space; at the end of this are naringenin and product analog-binding sites, and the space to the lower left of the end of the CoA-binding tunnel serves as the coumaroyl-binding pocket (Figures 8A,C). Speculated based on structure and previous literature residues of the CpCHS1 pocket (Ser 139, Glu 198, Thr 200, Thr 203, and Ser 350), which surround the coumaroyl-derived portion of the bound naringenin molecules, interact primarily through van der Waals contacts. And Phe 271 separates the coumaroyl-binding site from the cyclization pocket and may function as a mobile steric gate during successive rounds of polyketide elongation (Ferrer et al., 1999). Protein sequence alignment and structure analysis indicated that CHS proteins are highly conserved, and only a small modification of the active-site architecture leads to the remarkable functional diversity of the enzymes. When the structure of CpCHS1 is compared with the modeling structure of CpCHS2, portion residues of the naringenin pocket (Thr 138, Ser139, Leu 199, Thr 200, Val 202, Thr 203, and Ile260) are different (Figure 9B), indicating that these residues likely contribute to explain the different catalytic activity between CpCHS1 and CpCHS2. Thus, the CpCHS1 mutation results show that these residues, including T138S, S139G, L199T, T200I, V202I, T203F, and I260L, are critical to their enzymatic function, and single-point mutations analysis indicated that Leu199 and Thr203 were particularly important for producing naringenin from *p*-coumaroyl-CoA and three molecules of malonyl-CoA. These results provided the structural and biochemistry basis that the key amino acid of the active site principally influences the CpCHS1 functional diversity of *C. parasiticus*.

In conclusion, flavonoids play a variety of roles in various plants to resist biotic and abiotic stresses and possess a biological function as important dietary components and pharmaceutical value for human beings. From transcriptome, metabolome, to crystallography, we combined these powerful tools to investigate the flavonoids metabolic changes in plant different development stages and attempted to elucidate their detailed molecular mechanism. Our data provide a molecular basis for the flavonoids generating process, which may benefit the rational engineering of bacteria or yeast in synthetic biology that can then produce more meritorious compounds of flavonoids in the future.

## Data Availability Statement

The original contributions presented in the study are publicly available. This data can be found here: National Center for Biotechnology Information (NCBI) BioProject database under accession number PRJNA747631.

## Author Contributions

A-XC, JXL, PZ, and H-XL conceived the research plan and designed the experiments. MN, JF, RN, T-TZ, R-LX, and JXL performed the experiments and analyzed data. MN, JF, JXL, and A-XC wrote the paper. All authors read and approved the final manuscript.

## Funding

This work was funded by the National Key R&D Program of China (2018YFA0900602) and the National Natural Science Foundation of China (Nos. 31770330, 32000228, and 31870720). JXL is supported by the Foundation of Youth Innovation Promotion Association of the Chinese Academy of Sciences.

## Conflict of Interest

The authors declare that the research was conducted in the absence of any commercial or financial relationships that could be construed as a potential conflict of interest.

## Publisher's Note

All claims expressed in this article are solely those of the authors and do not necessarily represent those of their affiliated organizations, or those of the publisher, the editors and the reviewers. Any product that may be evaluated in this article, or claim that may be made by its manufacturer, is not guaranteed or endorsed by the publisher.

## References

[B1] Abe I. Morita H. (2010). Structure and function of the chalcone synthase superfamily of plant type III polyketide synthases. Nat. Prod. Rep. 27, 809–838. 10.1039/b909988n 20358127

[B2] Adams P. D. Afonine P. V. Bunkoczi G. Chen V. B. Davis I. W. Echols N.. (2010). PHENIX: a comprehensive Python-based system for macromolecular structure solution. Acta Crystallogr. D Biol. Crystallogr. 66, 213–221. 10.1107/S0907444909052925 20124702 PMC2815670

[B3] Butelli E. Titta L. Giorgio M. Mock H. P. Matros A. Peterek S.. (2008). Enrichment of tomato fruit with health-promoting anthocyanins by expression of select transcription factors. Nat. Biotechnol. 26, 1301–1308. 10.1038/nbt.1506 18953354

[B4] Chen S. Zhou Y. Chen Y. Gu J. (2018). fastp: an ultra-fast all-in-one FASTQ preprocessor. Bioinformatics 34, i884–i890. 10.1093/bioinformatics/bty560 30423086 PMC6129281

[B5] Cheng A. X. Zhang X. Han X. J. Zhang Y. Y. Gao S. Liu C. J.. (2018). Identification of chalcone isomerase in the basal land plants reveals an ancient evolution of enzymatic cyclization activity for synthesis of flavonoids. New Phytol. 217, 909–924. 10.1111/nph.14852 29083033

[B6] Dobrowolska I. Businge E. Abreu I. N. Moritz T. Egertsdotter U. (2017). Metabolome and transcriptome profiling reveal new insights into somatic embryo germination in Norway spruce (*Picea abies*). Tree Physiol. 37, 1752–1766. 10.1093/treephys/tpx078 28985382

[B7] Dong T. Han R. Yu J. Zhu M. Zhang Y. Gong Y.. (2019). Anthocyanins accumulation and molecular analysis of correlated genes by metabolome and transcriptome in green and purple asparaguses (*Asparagus officinalis* L.). Food Chem. 271, 18–28. 10.1016/j.foodchem.2018.07.120 30236664

[B8] Emsley P. Cowtan K. (2004). Coot: model-building tools for molecular graphics. Acta Crystallogr. D Biol. Crystallogr. 60, 2126–2132. 10.1107/S0907444904019158 15572765

[B9] Ferrer J. L. Jez J. M. Bowman M. E. Dixon R. A. Noel J. P. (1999). Structure of chalcone synthase and the molecular basis of plant polyketide biosynthesis. Nat. Struct. Biol. 6, 775–784. 10.1038/11553 10426957

[B10] Imaizumi R. Mameda R. Takeshita K. Kubo H. Sakai N. Nakata S.. (2020). Crystal structure of chalcone synthase, a key enzyme for isoflavonoid biosynthesis in soybean. Proteins 89, 126–131. 10.1002/prot.25988 32725893

[B11] Jez J. M. Austin M. B. Ferrer J. L. Bowman M. E. Noel J. P. J. C. C. B. (2000). Structural control of polyketide formation in plant-specific polyketide synthases. Chem. Biol. 7, 919–930. 10.1016/s1074-5521(00)00041-7 11137815

[B12] Jiang T. Zhang M. Wen C. Xie X. Tian W. Wen S.. (2020). Integrated metabolomic and transcriptomic analysis of the anthocyanin regulatory networks in Salvia miltiorrhiza Bge. flowers. BMC Plant Biol. 20:349. 10.1186/s12870-020-02553-7 32703155 PMC7379815

[B13] Li J. Yu F. Guo H. Xiong R. Zhang W. He F.. (2020). Crystal structure of plant PLDalpha1 reveals catalytic and regulatory mechanisms of eukaryotic phospholipase D. Cell Res. 30, 61–69. 10.1038/s41422-019-0244-6 31619765 PMC6951347

[B14] Liou G. Chiang Y. C. Wang Y. Weng J. K. (2018). Mechanistic basis for the evolution of chalcone synthase catalytic cysteine reactivity in land plants. J. Biol. Chem. 293, 18601–18612. 10.1074/jbc.RA118.005695 30291143 PMC6290136

[B15] Luceri C. Giovannelli L. Pitozzi V. Toti S. Castagnini C. Routaboul J. M.. (2008). Liver and colon DNA oxidative damage and gene expression profiles of rats fed *Arabidopsis thaliana* mutant seeds containing contrasted flavonoids. Food Chem. Toxicol. 46, 1213–1220. 10.1016/j.fct.2007.10.007 18035473

[B16] Martens S. Mithofer A. (2005). Flavones and flavone synthases. Phytochemistry 66, 2399–2407. 10.1016/j.phytochem.2005.07.013 16137727

[B17] Minor W. Cymborowski M. Otwinowski Z. Chruszcz M. (2006). HKL-3000: the integration of data reduction and structure solution—from diffraction images to an initial model in minutes. Acta Crystallogr. D Biol. Crystallogr. 62, 859–866. 10.1107/S0907444906019949 16855301

[B18] Morita H. Wong C. P. Abe I. (2019). How structural subtleties lead to molecular diversity for the type III polyketide synthases. J. Biol. Chem. 294, 15121–15136. 10.1074/jbc.REV119.006129 31471316 PMC6791334

[B19] Ni R. Zhu T. T. Zhang X. S. Wang P. Y. Sun C. J. Qiao Y. N.. (2020). Identification and evolutionary analysis of chalcone isomerase-fold proteins in ferns. J. Exp. Bot. 71, 290–304. 10.1093/jxb/erz425 31557291 PMC6913697

[B20] Reimold U. Kröger M. Kreuzaler F. Hahlbrock K. J. E. J. (1983). Coding and 3' non-coding nucleotide sequence of chalcone synthase mRNA and assignment of amino acid sequence of the enzyme. EMBO J. 2, 1801–1805. 16453477 10.1002/j.1460-2075.1983.tb01661.x PMC555362

[B21] Righini S. Rodriguez E. J. Berosich C. Grotewold E. Casati P. Falcone Ferreyra M. L. (2019). Apigenin produced by maize flavone synthase I and II protects plants against UV-B-induced damage. Plant Cell Environ. 42, 495–508. 10.1111/pce.13428 30160312

[B22] Sangpong L. Khaksar G. Pinsorn P. Oikawa A. Sasaki R. Erban A.. (2021). Assessing dynamic changes of taste-related primary metabolism during ripening of durian pulp using metabolomic and transcriptomic analyses. Front. Plant Sci. 12:687799. 10.3389/fpls.2021.687799 34220909 PMC8250156

[B23] Shomura Y. Torayama I. Suh D. Y. Xiang T. Kita A. Sankawa U.. (2005). Crystal structure of stilbene synthase from *Arachis hypogaea*. Proteins 60, 803–806. 10.1002/prot.20584 16028220

[B24] Sun W. Meng X. Liang L. Jiang W. Huang Y. He J.. (2015). Molecular and biochemical analysis of chalcone synthase from freesia hybrid in flavonoid biosynthetic pathway. PLoS ONE 10:e0119054. 10.1371/journal.pone.0119054 25742495 PMC4351062

[B25] Tamura K. Peterson D. Peterson N. Stecher G. Nei M. Kumar S. (2011). MEGA5: molecular evolutionary genetics analysis using maximum likelihood, evolutionary distance, and maximum parsimony methods. Mol. Biol. Evol. 28, 2731–2739. 10.1093/molbev/msr121 21546353 PMC3203626

[B26] Waterhouse A. Bertoni M. Bienert S. Studer G. Tauriello G. Gumienny R.. (2018). SWISS-MODEL: homology modelling of protein structures and complexes. Nucleic Acids Res. 46, W296–W303. 10.1093/nar/gky427 29788355 PMC6030848

[B27] Wei H. Zhang X. Wu G. Yang X. Pan S. Wang Y.. (2013). Chalcone derivatives from the fern *Cyclosorus parasiticus* and their anti-proliferative activity. Food Chem. Toxicol. 60, 147–152. 10.1016/j.fct.2013.07.045 23891701

[B28] Winkel-Shirley Physiology BJP. (2001). Flavonoid biosynthesis. A colorful model for genetics, biochemistry, cell biology, and biotechnology. Plant Physiol. 126, 485–493. 10.1104/pp.126.2.485 11402179 PMC1540115

[B29] Xie Y. Zheng Y. Dai X. Wang Q. Cao J. Xiao J. (2015). Seasonal dynamics of total flavonoid contents and antioxidant activity of *Dryopteris erythrosora*. Food Chem. 186, 113–118. 10.1016/j.foodchem.2014.05.024 25976799

[B30] Yonekura-Sakakibara K. Higashi Y. Nakabayashi R. (2019). The origin and evolution of plant flavonoid metabolism. Front. Plant Sci. 10:943. 10.3389/fpls.2019.00943 31428108 PMC6688129

[B31] Yu H. N. Liu X. Y. Gao S. Sun B. Zheng H. B. Ji M.. (2018). Structural and biochemical characterization of the plant type III polyketide synthases of the liverwort Marchantia paleacea. Plant Physiol. Biochem. 125, 95–105. 10.1016/j.plaphy.2018.01.030 29428820

[B32] Yu H. N. Wang L. Sun B. Gao S. Cheng A. X. Lou H. X. (2015). Functional characterization of a chalcone synthase from the liverwort *Plagiochasma appendiculatum*. Plant Cell Rep. 34, 233–245. 10.1007/s00299-014-1702-8 25404490

[B33] Zhang X. Gou M. Liu C. J. (2013). Arabidopsis Kelch repeat F-box proteins regulate phenylpropanoid biosynthesis *via* controlling the turnover of phenylalanine ammonia-lyase. Plant Cell 25, 4994–5010. 10.1105/tpc.113.119644 24363316 PMC3904001

[B34] Zhang X. Wang X. Wang M. Cao J. Xiao J. Wang Q. (2019). Effects of different pretreatments on flavonoids and antioxidant activity of *Dryopteris erythrosora* leave. PLoS ONE 14:e0200174. 10.1371/journal.pone.0200174 30601805 PMC6314590

[B35] Zhang Y. Hu Z. Chu G. Huang C. Tian S. Zhao Z.. (2014). Anthocyanin accumulation and molecular analysis of anthocyanin biosynthesis-associated genes in eggplant (*Solanum melongena* L.). J. Agric. Food Chem. 62, 2906–2912. 10.1021/jf404574c 24654563

