# Supplementary material for: Functional and Structural Investigation of Chalcone Synthases Based on Integrated Metabolomics and Transcriptome Analysis on Flavonoids and Anthocyanins Biosynthesis of the Fern Cyclosorus parasiticus
Source: Front Plant Sci. 2021 Oct 28;12:757516. doi: 10.3389/fpls.2021.757516 (PMC8580882; doi:10.3389/fpls.2021.757516)
Supplement: Supplementary file 1 [file Data_Sheet_1.PDF]

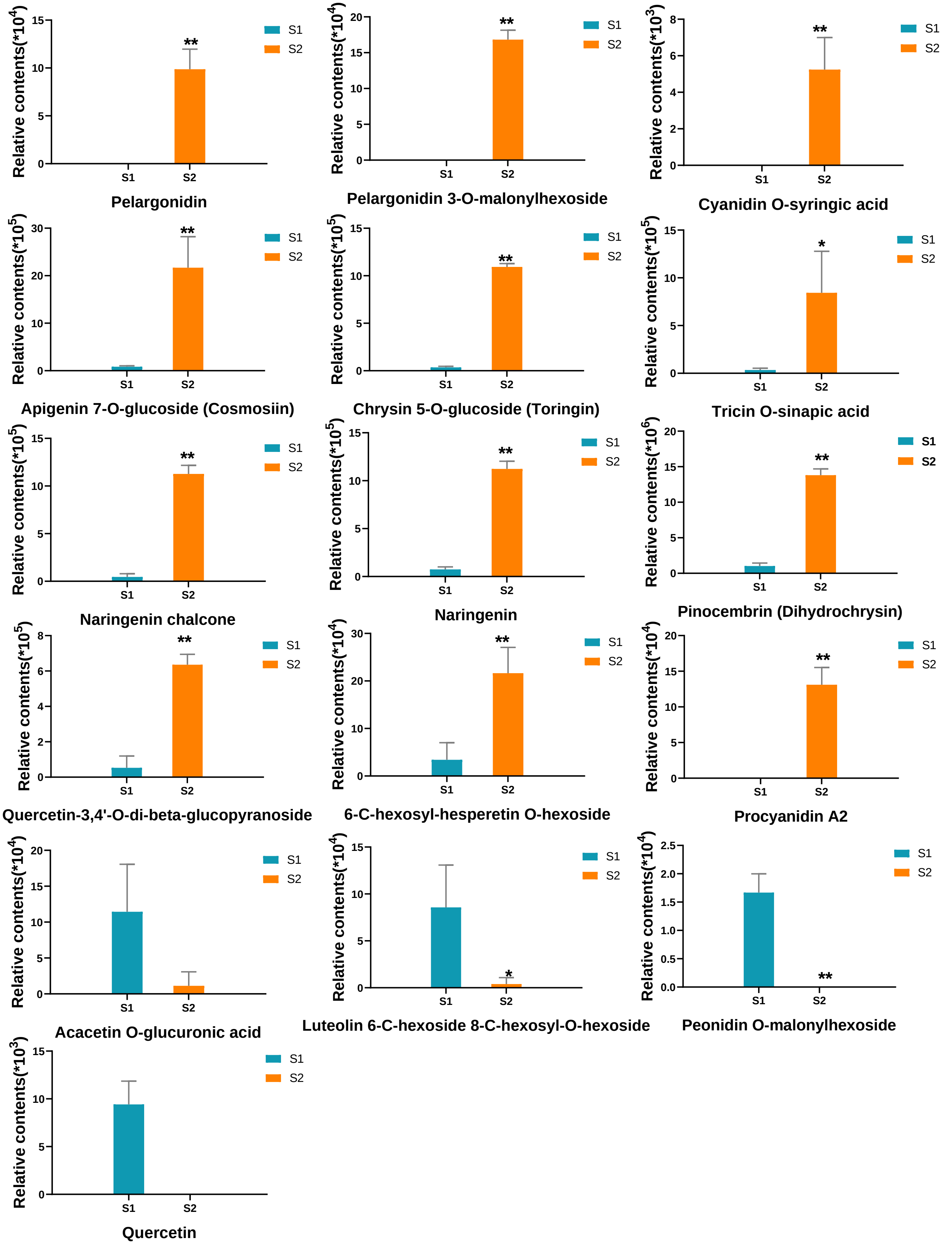

Supplementary Figure 1 Relative contents of flavonoid and anthocyanin metabolites in S1 and S2. Data are means ±SD of three biological replicates. Statistical analysis was performed with Student's *t*-test. *, **: means differ from the S1 at $p < 0.05$ and $p < 0.01$, respectively.

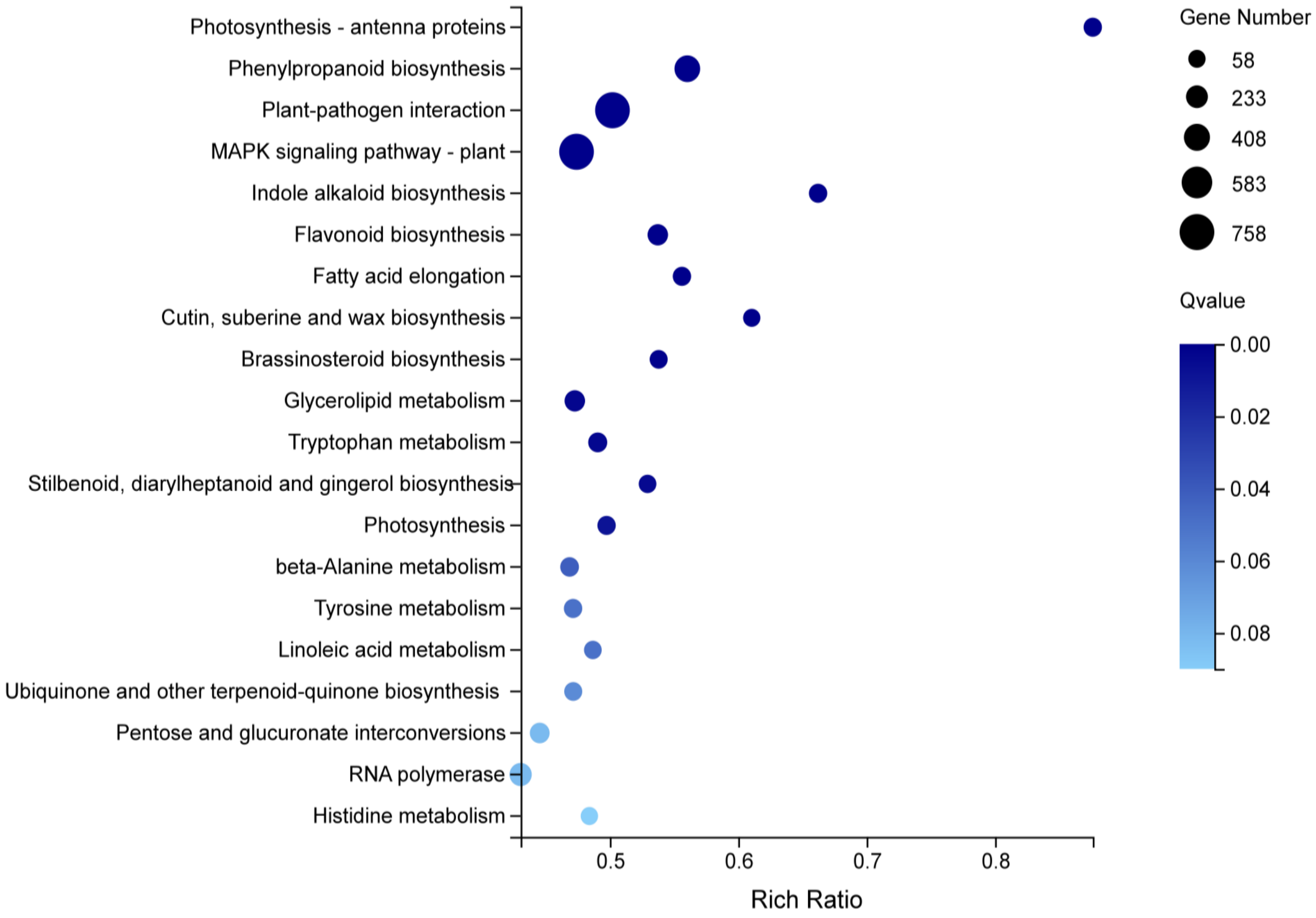

Supplementary Figure 2 KEGG pathway enrichment of differently expressed genes. The size of the black diamonds mean the gene number and the different color represent the Q-value.

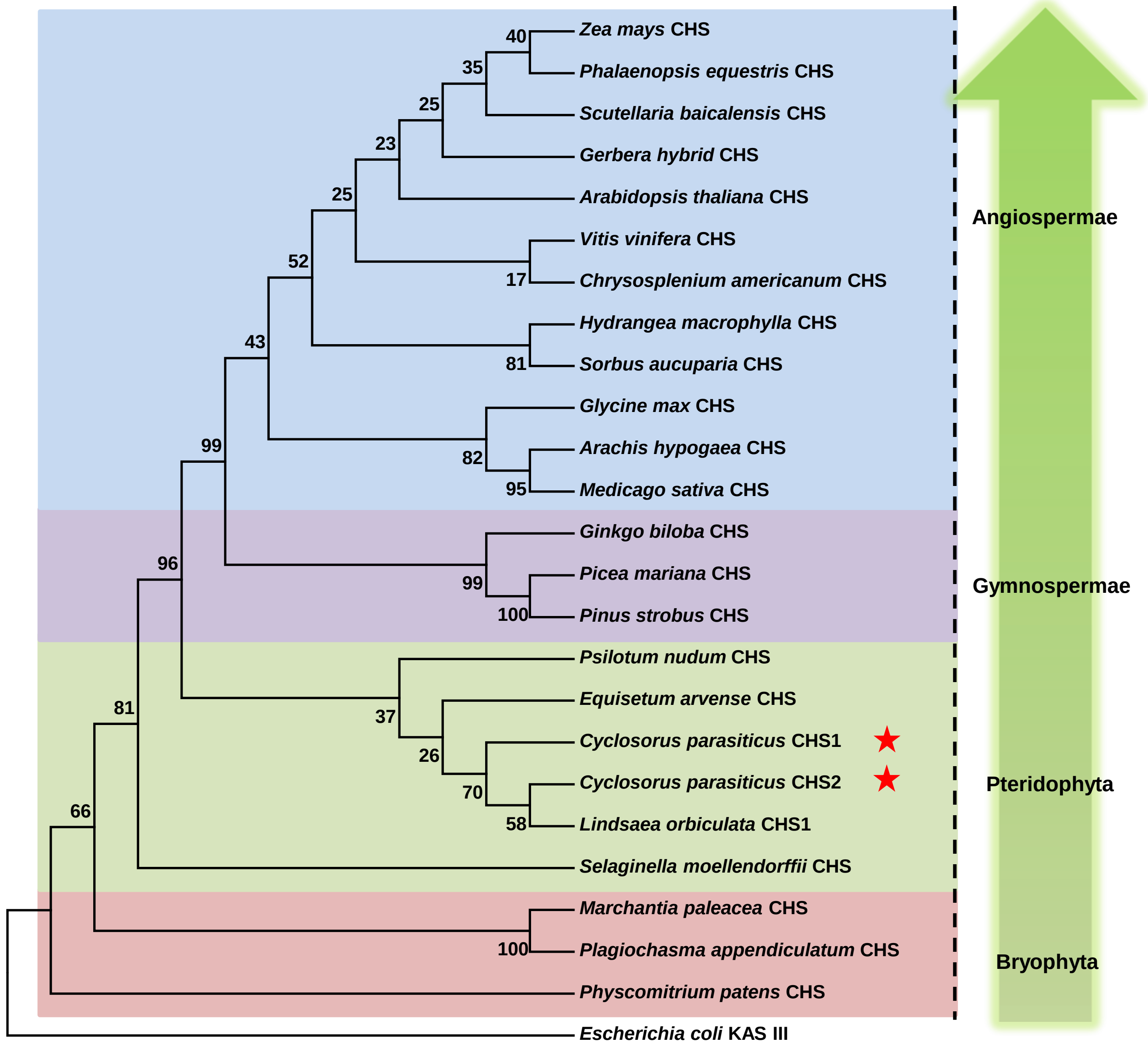

Supplementary Figure 3 Phylogeny of plant CHS. The tree was constructed using the neighbor-joining method applying 1000 bootstrap replicates. Bootstrap values are shown as percentages next to branch points. The length of each branch reflects the distance between nodes. The four plant families are highlighted with different color boxes, respectively. The sequences used include *Hydrangea macrophylla* CHS (BAA32732), *Sorbus aucuparia* CHS (ABB89213), *Vitis vinifera* CHS (CAA53583), *Arabidopsis thaliana* CHS (AT5G13930), *Chrysosplenium americanum* CHS (AAB54075), *Gerbera hybrid* CHS (CAA86218), *Scutellaria baicalensis* CHS (BAA23373), *Zea mays* CHS (CAA42764), *Phalaenopsis equestris* CHS (AIS35912.1), *Glycine max* CHS (ABB30178), *Arachis hypogaea* CHS (AAO32821), *Medicago sativa* CHS (AAB41559.1), *Ginkgo biloba* CHS (AAT68477), *Picea mariana* CHS (AAF35890), *Pinus strobus* CHS (CAA06077), *Lindsaea orbiculata* CHS1 (QDF63003.1), *Psilotum nudum* CHS (BAA87922), *Equisetum arvense* CHS (Q9MBB1.1), *Marchantia paleacea* CHS (KY968327.1), *Huprezia serrata* CHS (ABI94386), *Selaginella moellendorffii* CHS (6DX8), *Physcomitrium patens* CHS (ABB84527.1), *Plagiochasma appendiculatum* CHS (KM506763.1), *E. coli* β-ketoacyl-(acyl carrier protein) synthase III (KAS III, BAA35899) served as an outgroup.

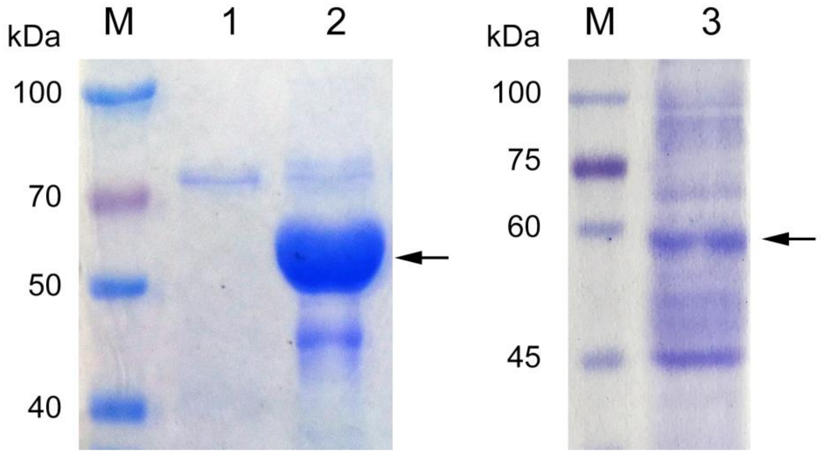

Supplementary Figure 4 SDS-PAGE separation of recombinant proteins: CpCHS1, CpCHS2. M: molecular mass standards; Lane 1: proteins purified from pET32a; lanes 2: proteins purified from CpCHS1-pET32a; lanes 3: proteins purified from CpCHS2-pET32a.

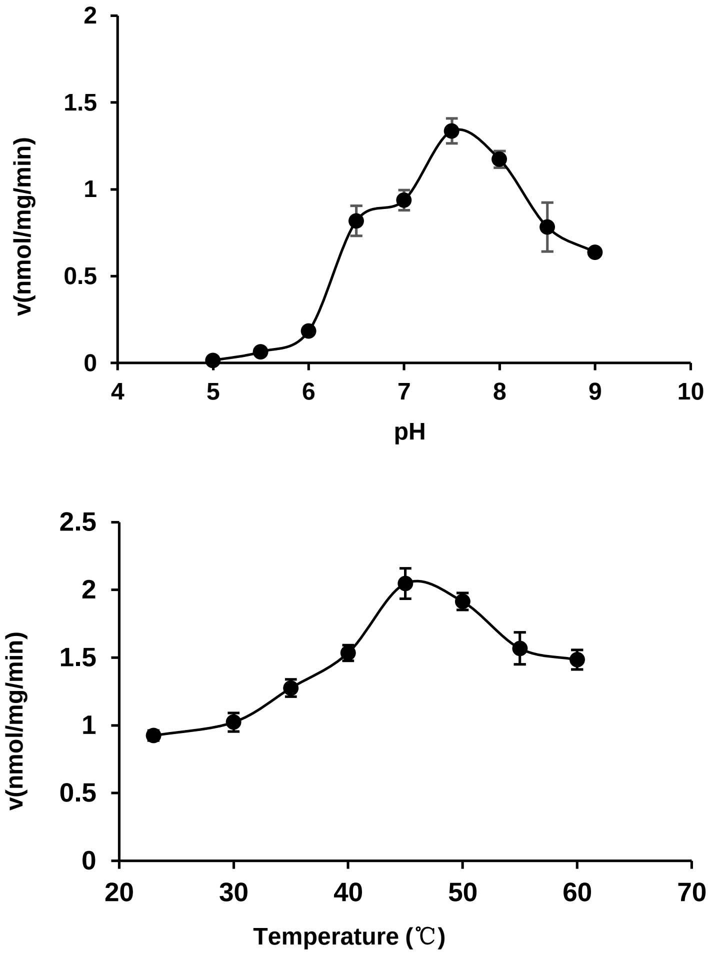

Supplementary Figure 5 The catalytic activity of the CpCHS1 with different pH and temperature.

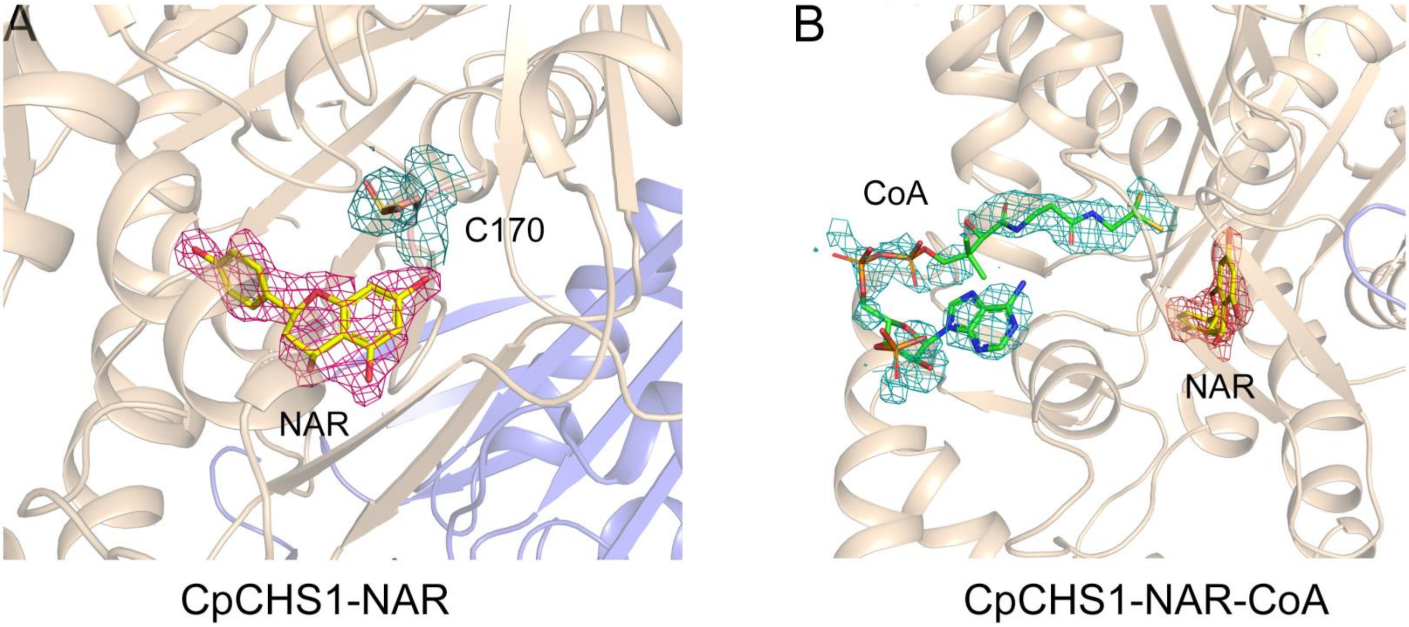

Supplementary Figure 6 Electron densities of CpCHS1 complex structure. (**A**) Electron densities of CpCHS1- NAR; (**B**) Electron densities of CpCHS1- NAR-CoA..

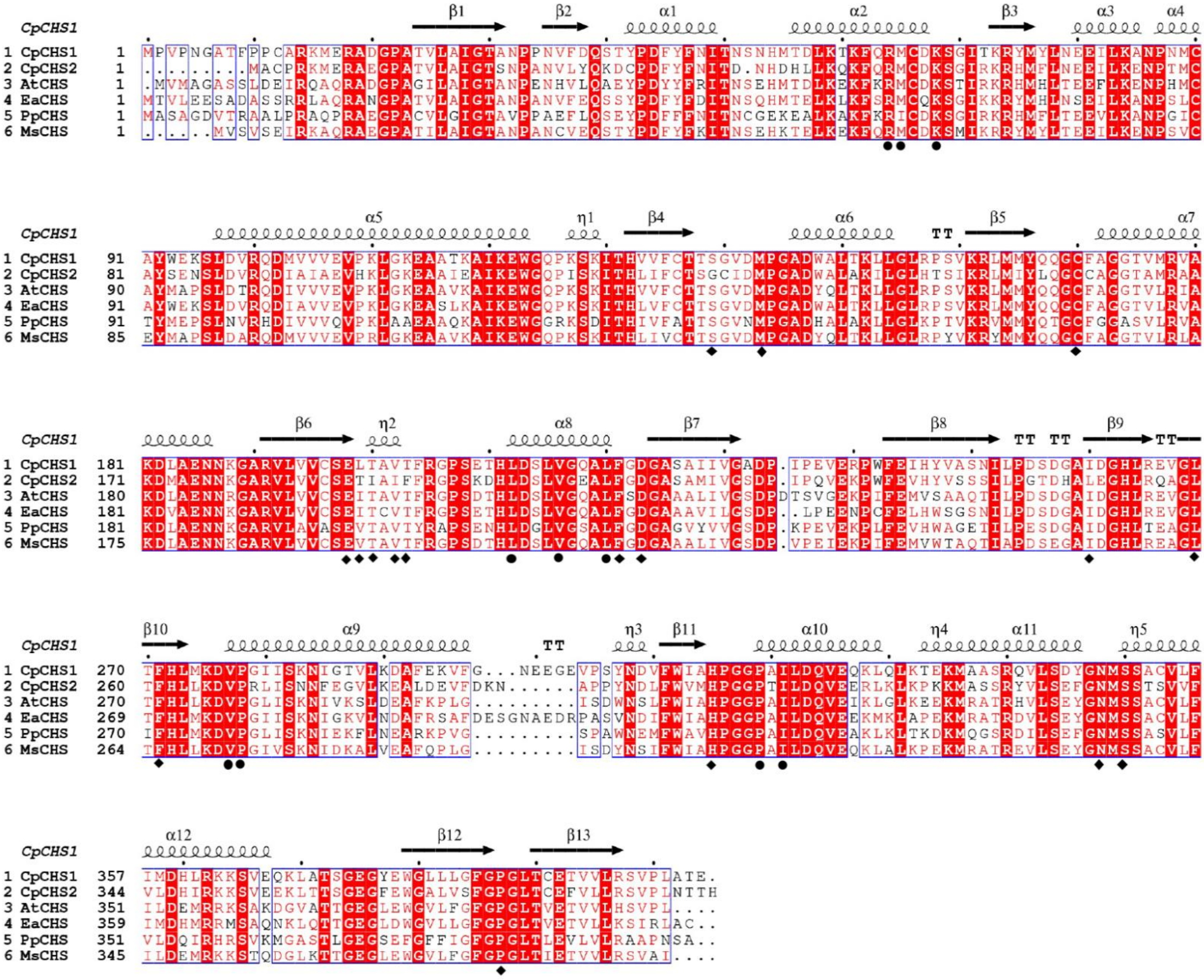

Supplementary Figure 7 Sequence alignment analysis of CpCHS1, CpCHS2, AtCHS (*A. thaliana* CHS, AT5G13930), EaCHS (*E. arvense* CHS, Q9MBB1.1), PpCHS (*P. patens* CHS, ABB84527) and MsCHS (*M. sativa* CHS, AAB41559.1). Black diamonds mean the key residues of naringenin binding pocket; Black dots mean the key residues of CoA binding pocket.

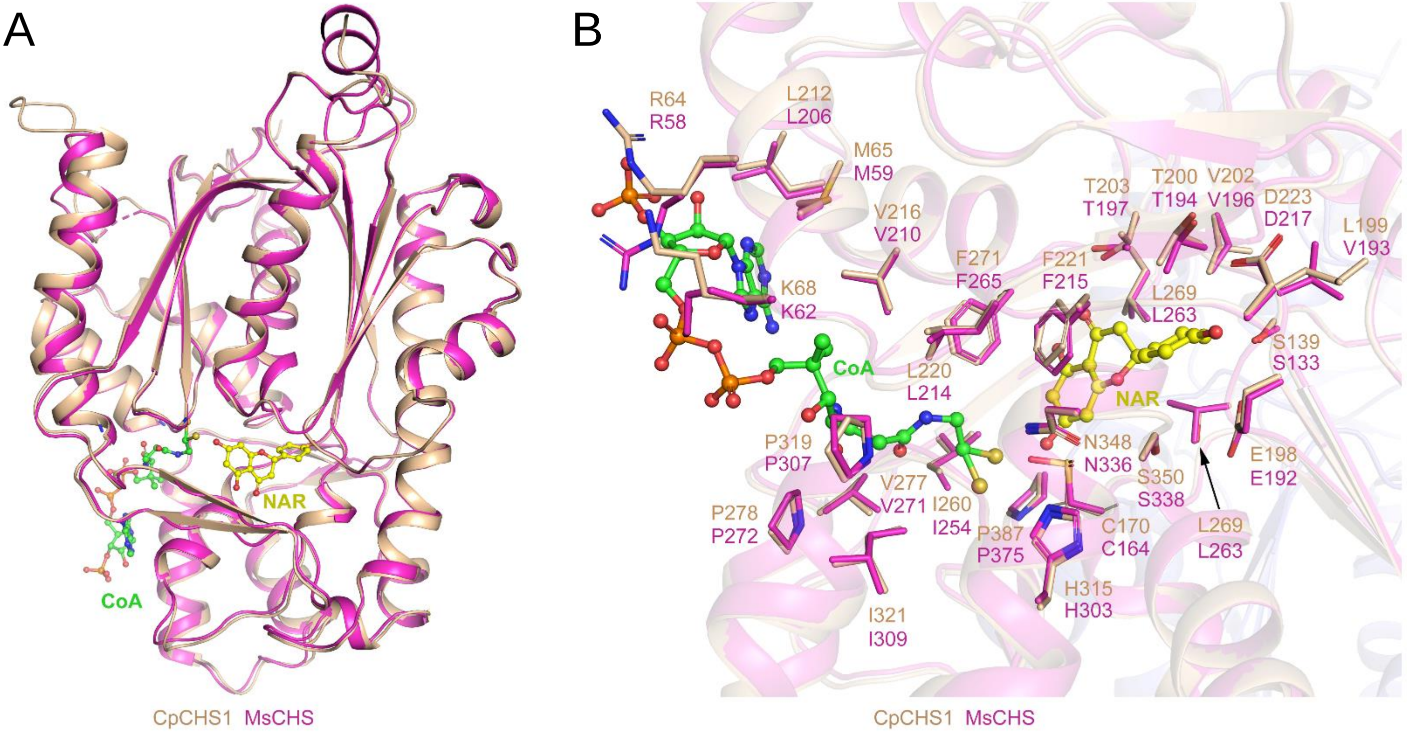

Supplementary Figure 8 Structure comparison analysis of CpCHS1 and MsCHS (PDB: 1CGK). (**A**) The overall structure superimposition of CpCHS1 and MsCHS; (**B**) Comparison of the amino acid residues that form the substrate binding pocket of CpCHS1 and MsCHS.

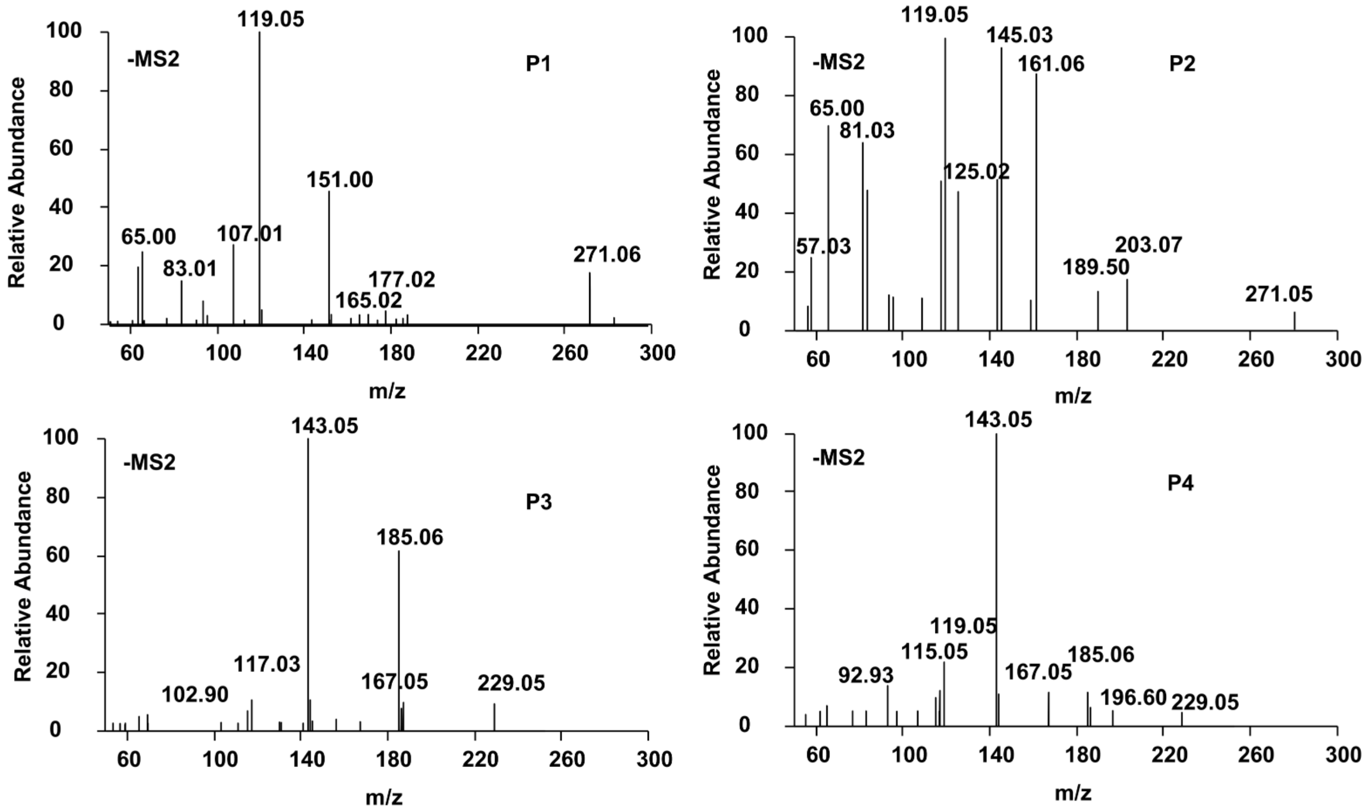

Supplementary Figure 9 Mass spectrum analysis of products generated by the CpCHS1 wild type and CpCHS1 mutant using *p*-coumaroyl-CoA as substrates, P1: naringenin, P2: CTAL, P3, P4: BNY and isomer of BNY.
