# Supplementary material for: Functional and Structural Investigation of Chalcone Synthases Based on Integrated Metabolomics and Transcriptome Analysis on Flavonoids and Anthocyanins Biosynthesis of the Fern Cyclosorus parasiticus
Source: Front Plant Sci. 2021 Oct 28;12:757516. doi: 10.3389/fpls.2021.757516 (PMC8580882; doi:10.3389/fpls.2021.757516)
Supplement: Supplementary file 2 [file Data_Sheet_2.docx]

**Supplementary Table 1** Sequences of specific primers for qRT-PCR.

| **Primer name** | **Forward (5'-3')** | | **Reverse (5'-3')** |
| --- | --- | --- | --- |
| F3'H (CL1149.Contig3) | GGCTATGACATCCCTCCTAA | | GGCACCCTCTTCTACCAGAT |
| C4H (Unigene11283) | | CCTTGTTGTTGTATCGTCCC | CAATAAACCCTTGACTGCTG |
| DFR (CL871.Contig7) | CAGGTGAATCTGAGCATTTC | | AGGCTTCCAAACTCTGTTG |
| 4CL (CL1096.Contig5) | GGCATCGGTCTCTTACAAT | | GGAGGAACCTGATAGCCTT |
| CHS (CL5031.Contig1) | TGGCACGGTTATGAGGGTTGCA | | TGGCACCATCACTATCCGGCA |
| PAL (CL5392.Contig1) | TCGTCGCATGATCCGCCAGT | | ATCGACGCGGGGTTTGGCTT |
| ANS (CL787.Contig1) | AGGATTCACATAGAGAGGAG | | TACTCATCCATTGTCTCCCT |
| F3H (CL3598.Contig1) | TAGAGAGGAGATTGTTCAGC | | CTGGAGAAAGAGGAACAAGT |
| Actin (Unigene10363) | GCCACATGCCATCCTTCGTCT | | ACAAGACCTCCGGACACCTGA |

| **Supplementary Table 2** Sequences of specific primers for PCR. | |
| --- | --- |
| **Primer name** | **Primer sequences 5'-3'** |
| CpCHS1-F | CTGTGGGCTTAGTTTTCTCT |
| CpCHS1-R | TGATAGGTTTTTGCTGTTGC |
| CpCHS1-BamHI-F | CGGGATCCATGCCGGTCCCTAATGGTGC |
| CpCHS1-XhoI-R | CCCTCGAGCTACTCGGTAGCAAGAGGCA |
| CpCHS2-F-1 | TCTCTCTCTGTGTGTGTGTG |
| CpCHS2-R-1 | ACACACACACACACACACAC |
| CpCHS2-F-2 | TAGAGAGCGGGTAGATAGAG |
| CpCHS2-R-2 | CTTCCATCTTATTTTGTGGG |
| CpCHS2-BamHI-F | CGGGATCCATGGCATGTCCTCGCAAGAT |
| CpCHS2-EcoRI-R | CGGAATTCTTAGTGAGTTGTATTGAGAG |

**Supplementary Table 3** Sequences of specific primers for mutant PCR.

| **Primer name** | **Primer sequences 5'-3'** |
| --- | --- |
| CpCHS1-S-BamHI | TCATCA CCACAGCCAGGATCCG ATGCCGGTCCCTAATGGTG |
| CpCHS1-AS-NotI | CGACTTAAGCATTATGCGGCCGC CTACTCGGTAGCAAGAGGCACAC |
| CpCHS1-S-T138S | GTTTTCTGCACTTCCAGTGGCGTTG |
| CpCHS1-AS-T138S | CAACGCCACTGGAGTGCAGAAAAC |
| CpCHS1-S-S139G | CTGCACTACCGGTGGCGTTGATATG |
| CpCHS1-AS- S139G | CATATCAACGCCACCGGTAGTGCAG |
| CpCHS1-S-L199T | GTGCAGTGAGACAACTGCTGTGAC |
| CpCHS1-AS- L199T | GTCACAGCAGTTGTCTCACTGCAC |
| CpCHS1-S-T200I | GCAGTGAGCTAATTGCTGTGACTTTCC |
| CpCHS1-AS-T200I | GGAAAGTCACAGCAATTAGCTCACTGC |
| CpCHS1-S-V202I | GCTAACTGCTATCACTTTCCGGGG |
| CpCHS1-AS-V202I | CCCCGGAAAGTGTAGCAGTTAGC |
| CpCHS1-S-T203F | GCTAACTGCTGTGTTTTTCCGGGGACC |
| CpCHS1-AS- T203F | GGTCCCCGGAAAAACACAGCAGTTAGC |
| CpCHS1-S-I260L | GTGATGGTGCCCTTGATGGACATC- |
| CpCHS1-AS-I260L | GATGTCCATCAAGGGCACCATCAC |
| CpCHS1-S-199+203 | GTGCAGTGAGACAACTGCTGTG TTTTTCCGGGGACC |
| CpCHS1-AS-199+203 | GGTCCCCGGAAAAACACAGCAGTTGTCTCACTGCAC |
| CpCHS1-S-138-139 | GTTTTCTGCACTTCCGGTGGCGTTGATATG |
| CpCHS1-AS-138-139 | CATATCAACGCCACCGGAAGTGCAGAAAAC |
| CpCHS1-S-199-203 | GTGCAGTGAGACAATTGCTATCTTTTTCCGGGGACC |
| CpCHS1-AS-199-203 | GGTCCCCGGAAAAAGATAGCAATTGTCTCACTGCAC |

**Supplementary Table 4** Data collection and structure refinement statistics.

|  | CpCHS1-Apo | CpCHS1-Nar | CpCHS1-Nar-CoA |
| --- | --- | --- | --- |
| Resolution (Å) | 39.64 - 1.9  (1.968 - 1.9) | 30.16- 2.397  (2.483 - 2.397) | 32.48 -2.399  (2.485 - 2.399) |
| Space group | P 2_1_ 2_1_ 2_1_ | P _1_ 2_1_ _1_ | P _1_ 2_1_ _1_ |
| Until cell |  | | |
| a, b, c, (Å) | 71.155, 95.462, 246.739 | 72.259, 82.289, 134.312 | 74.664, 82.893, 143.161 |
| α, β, γ, (°) | 90, 90, 90 | 90, 98.942, 90 | 90, 103.341, 90 |
| Unique reflections | 131165 (12527) ^†^ | 58380 (4711) | 63633 (5215) |
| Multiplicity | 12.4 | 6.7 | 6.6 |
| I/σ(I) | 11.0 (2.8) | 13.2 (2.8) | 15.1 (2.5) |
| Rmerge | 0.219 | 0.15 | 0.176 |
| Completeness (%) | 99.4 (99.0) | 100 (100) | 99.9 (100) |
| R_work_/R_free_‡ | 0.1816/0.2082 | 0.1803/0.2296 | 0.1791/0.2246 |
| Number of atoms | 13325 | 12224 | 12820 |
| Ligand |  | | |
| Nar | - | 80 | 80 |
| CoA | - | - | 147 |
| Water | 1099 | 185 | 694 |
| Protein residues | 1589 | 1564 | 1579 |
| r.m.s. deviations |  | | |
| Bonds (Å) | 0.009 | 0.014 | 0.012 |
| Angles (°) | 0.95 | 1.25 | 1.23 |
| Average B factor | 8.97 | 8.88 | 22.12 |
| Protein | 8.76 | 8.85 | 21.69 |
| Ligand | - | 9.46 | 42.31 |
| Water | 11.37 | 11.26 | 23.47 |
| Ramachandran |  |  |  |
| Favored (%) | 97.3 | 96.3 | 96.9 |
| Allowed (%) | 2.7 | 3.7 | 3.1 |

† Numbers in parentheses represent the highest-resolution shell.

‡ R =Σhkl||Fo|−|Fc||/Σhkl|Fo|.
